# Highly Efficient and Scalable p-i-n Perovskite Solar Cells Enabled by Poly-metallocene Interfaces

**DOI:** 10.1021/jacs.4c02220

**Published:** 2024-05-01

**Authors:** Bo Li, Danpeng Gao, Stephanie A. Sheppard, William D. J. Tremlett, Qi Liu, Zhen Li, Andrew J. P. White, Ryan K. Brown, Xianglang Sun, Jianqiu Gong, Shuai Li, Shoufeng Zhang, Xin Wu, Dan Zhao, Chunlei Zhang, Yan Wang, Xiao Cheng Zeng, Zonglong Zhu, Nicholas J. Long

**Affiliations:** † Department of Chemistry, City University of Hong Kong, Kowloon 999077, Hong Kong; ‡ Department of Chemistry, Imperial College London, MSRH Building, White City Campus, London W12 0BZ, U.K.; § Department of Materials Science & Engineering, City University of Hong Kong, Kowloon 999077, Hong Kong

## Abstract

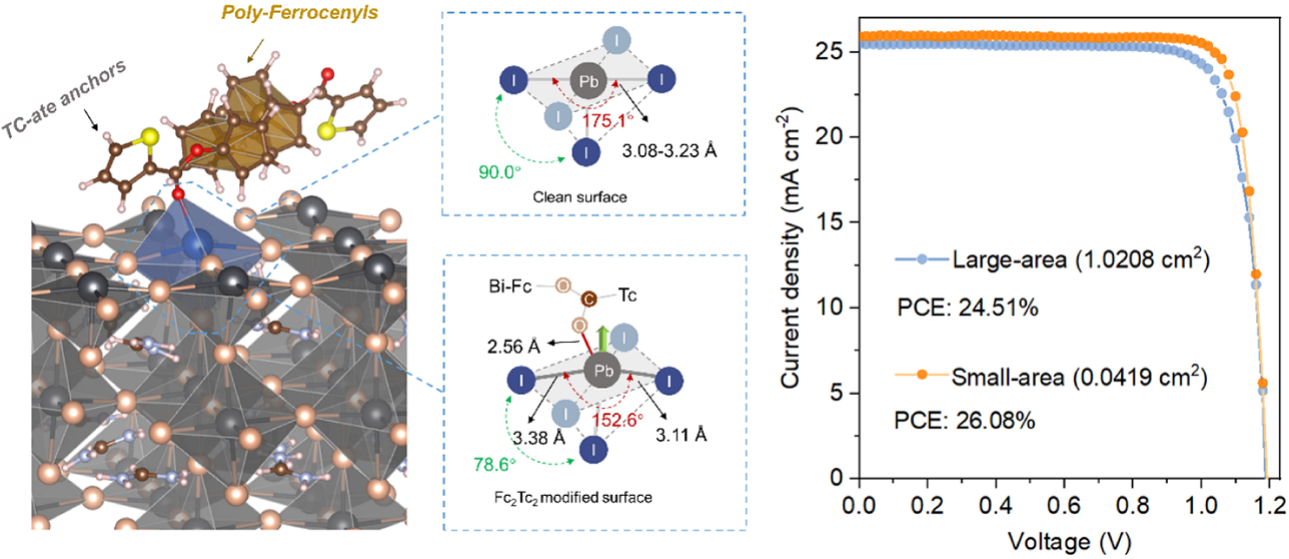

Inverted p-i-n perovskite solar cells (PSCs) are easy to process but need improved interface characteristics with reduced energy loss to prevent efficiency drops when increasing the active photovoltaic area. Here, we report a series of poly ferrocenyl molecules that can modulate the perovskite surface enabling the construction of small- and large-area PSCs. We found that the perovskite–ferrocenyl interaction forms a hybrid complex with enhanced surface coordination strength and activated electronic states, leading to lower interfacial nonradiative recombination and charge transport resistance losses. The resulting PSCs achieve an enhanced efficiency of up to 26.08% for small-area devices and 24.51% for large-area devices (1.0208 cm^2^). Moreover, the large-area PSCs maintain >92% of the initial efficiency after 2000 h of continuous operation at the maximum power point under 1-sun illumination and 65 °C.

## Introduction

Inverted p-i-n perovskite solar cells (PSCs) are rapidly advancing in their efficiencies and have shown potential for large-area module fabrication due to facile low-temperature processing and excellent operational stability.^1−10^ However, reducing efficiency losses on scaling up devices from small- to large-area remains a challenge for commercialization.^11−16^ The increase in area imposes higher demands on interface characteristics on a square centimeter scale.^17−20^ This is because nonradiative recombination typically occurs at the interface between the charge transport layers and perovskite terminations with high trap-state density, resulting in increased dark currents that limit the open-circuit voltage (*V*_OC_) and fill factor (FF) of the device.^21−27^ This limitation is especially challenging for large-area PSCs, where losses also originate from the inhomogeneity of the interfaces between the perovskite absorber layer and the transport layers, as well as from the transport resistance induced FF loss.^28^ Therefore, exploring effective approaches to improve interface characteristics to simultaneously enhance and homogenize the photovoltaic performance is crucial for the scalable fabrication of inverted PSCs.

**Figure 1:**
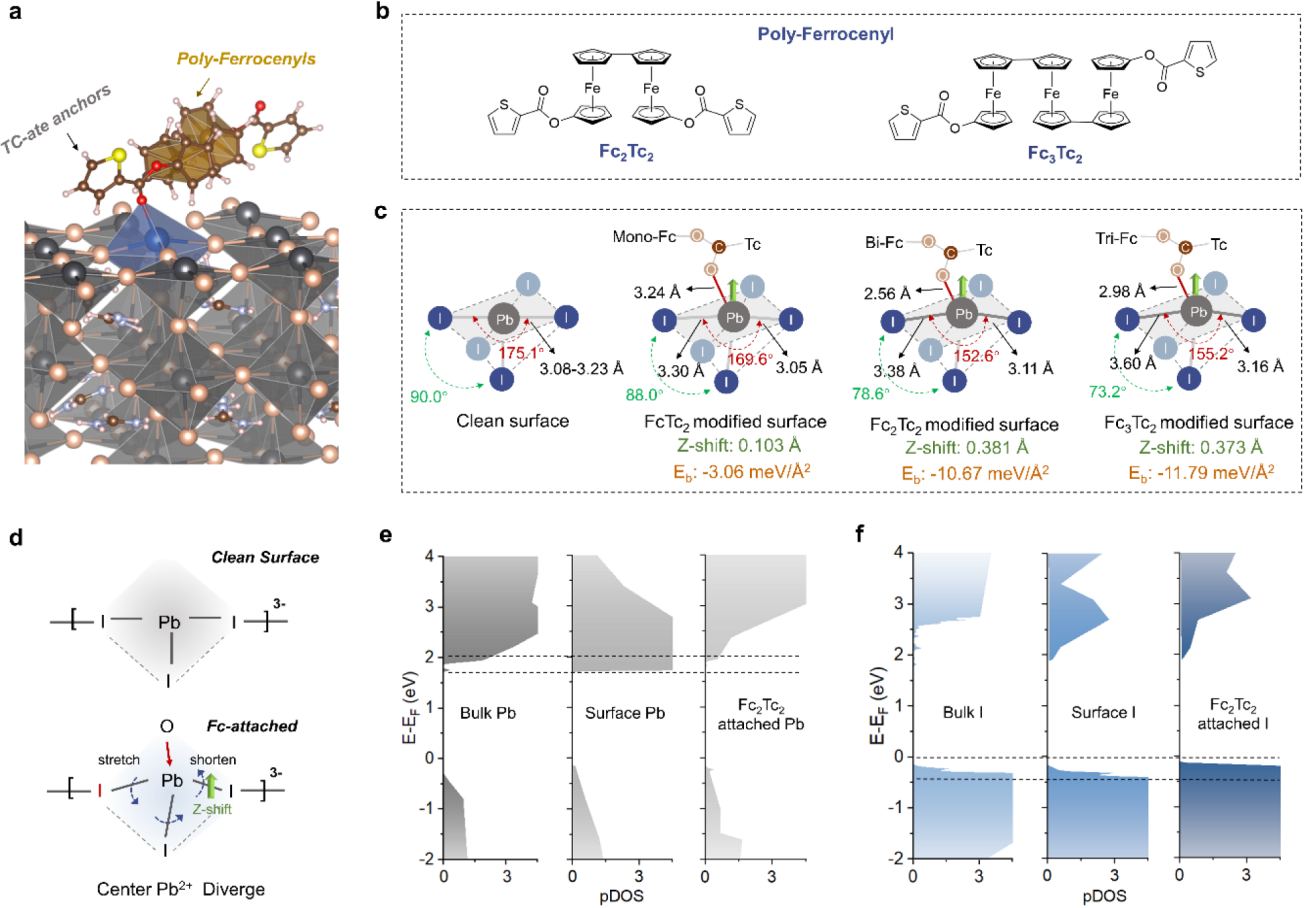
Surface molecule interaction. (a) Schematic illustration of the structure of a Fc_2_Tc_2_-modified perovskite surface. (b) Structures of Fc_2_Tc_2_ and Fc_3_Tc_2_. (c) Perovskite surface crystal structure changes after attached by FcTc_2_, Fc_2_Tc_2_, and Fc_3_Tc_2_ molecules. The “*Z*-shift” indicates that the FcTc-attached Pb ion is uplifted from the perovskite surface in the *Z* direction, while the *E*_b_ represents the calculated surface binding energy. (d) Comparison of the coordination structures of clean perovskite surface and FcTc-attached surface. (e) pDOS plots of bulk Pb, surface Pb, and Fc_2_Tc_2_-coordinated Pb (f) pDOS plots of bulk I, surface I, and Fc_2_Tc_2_-coordinated I via DFT computation. FAPbI_3_ was used for the computational modeling of perovskites.

Following on from our 2022 report,^7^ we herein show a rational design strategy to optimize the interface of p-i-n PSCs based on a poly-ferrocenyl-modulated perovskite surface layer with reduced nonradiative recombination loss and transport resistance, leading to efficient and scalable PSCs. The hybrid complex [PbI_4_←Fc_n_Tc_2_]^3–^ (*n* = 1, 2, or 3) results in strong local geometrical distortion and activated electronic states on the perovskite surface, which greatly reduces surficial defect density and accelerates interfacial charge transfer. Through interface modification via biferrocenyl-bis-thiophene-2-carboxylate (Fc_2_Tc_2_; Figure 1b), we achieved an improved efficiency of 26.08% for small-area devices and 24.51% for large-area devices (1.0208 cm^2^). Moreover, the large-area PSCs maintain >92% of their initial efficiency after running continuously for 2000 h at the MPP and 65 °C under AM1.5 sunlight followed by the ISOS-L-2I protocol.

## Results and Discussion

### Surface Activation by Polyferrocenes

Figure 1a shows a schematic illustration of the perovskite surface modified with Fc_2_Tc_2_. Synthesis and structural and electrochemical characterization of poly-ferrocenyl molecules Fc_2_Tc_2_ and triferrocenyl-bis-thiophene-2-carboxylate (Fc_3_Tc_2_; Figure 1b) are summarized in Notes S1 and S2 and Figures S1–S17. We first performed density functional theory (DFT) calculations to study the electronic structure of the perovskite surface upon the introduction of poly-ferrocenyl molecules by Vienna Ab initio Simulation Package (VASP) 6.4. All DFT calculations are based on Perdew–Burke–Ernzerhof (PBE) functional with spin–orbit coupling (SOC) effect.^29−31^ The DFT-D3 dispersion correction of Grimme with zero damping was used for hydrogen bond optimization.^32−34^ A representative PbI_2_-termination with the (001) facet of perovskite was selected as the study object due to its low surface energy, which is dominant in perovskite crystal growth and more conducive to the binding of ferrocenyl molecules.^35,36^ Furthermore, the PbI_2_-rich condition in the precursor can enhance the PbI_2_-terminal formation and the PbI_2_ accumulation at the grain boundaries and surfaces of perovskite films during the crystallization process.^37,38^

As shown in Figures 1c and S18, the Pb–I bond lengths at the clean surface range from 3.08 to 3.23 Å. After modifying with ferrocenyl compounds, the carbonyl O atom bonds to the undercoordinated Pb^2+^ on the perovskite surface, and the Pb–I bond lengths at the stretched side change to 3.30, 3.38, and 3.60 Å, respectively, suggesting interactions between the ferrocenyl compounds and the perovskite (verified experimentally by X-ray photoelectron spectroscopy as shown in Figures S19 and S20). Ferrocenyl-bis-thiophene-2-carboxylate (FcTc_2_) coordinates with the perovskite surface [PbI_5_]^3–^ with a Pb–O bond length of 3.24 Å. Fc_2_Tc_2_ and Fc_3_Tc_2_ both have shorter Pb–O lengths than FcTc_2_, which are 2.56 and 2.98 Å, respectively. Moreover, the Tc-attached Pb is lifted further from the bottom center of the [PbI_5_]^3–^ pyramid, with a stronger bond length distortion than the clean surface (see the bond length variations in Figure 1c). The strong stereochemical blocking effect caused by Fc_2_Tc_2_ or Fc_3_Tc_2_ leads to surficial [PbI_5_]^3–^ being distorted away from the octahedral center due to stronger Pb–O interactions. It should be noted that the interaction does not affect the optical properties and morphologies of the perovskite films, as evidenced in Figures S21–S23.

We further calculated the surface binding energies of the three structures. It shows that FcTc_2_ has an *E*_b_ of −3.06 meV/Å^2^, while the poly ferrocenyl complexes behave with lower *E*_b_ values of −10.67 meV/Å^2^ (Fc_2_Tc_2_) and −11.79 meV/Å^2^ (Fc_3_Tc_2_). Consequently, for Fc_2_Tc_2_ in Figure 1d, the Pb–I bond inside the distorted [PbI_5_]^3–^ is alternately stretched and shortened, and the original [PbI_5_]^3–^ is transformed into an FcTc-coordinated hybrid complex in the form of [PbI_4_←Fc_2_Tc_2_]^3–^, where the more strongly stretched Pb–I bond contributes to the elevation of the VBM level. For Fc_3_Tc_2_, the geometric blocking effect leads to more structural differences from Fc_2_Tc_2_, since the rotational freedom of the Fc moieties results in Fc_3_Tc_2_ to adopt a more disordered conformation than Fc_2_Tc_2_. The more conformation freedom of Fc_3_Tc_2_ results in less interaction with the perovskite surface (See Figure S18). Thus, we ruled out Fc_3_Tc_2_ in the electronic structure simulation.

This coordination effect yields strong interactions between the surface Pb and terminal-Tc carbonyl group, which leads to reduced Pb-6p contribution and enhanced I-5p contribution, due to the destruction of the surface [PbI_5_]^3–^ framework, as illustrated in Figure S24. The projection density of states (pDOS) of Pb in Figure 1e reveals that the surface Pb has a lower conduction band minimum (CBM) by 0.2 eV than the bulk Pb, due to the undercoordinated orbitals of Pb atoms in the surface [PbI_5_]^3–^. The coordination of Fc_2_Tc_2_ shifts the CBM of the surface Pb to the same energy level as the bulk Pb, indicating the electronic configuration is restored closely to [PbI_6_]^4–^ in the bulk phases, which confirms the passivation effect of Fc_2_Tc_2_ toward the surface Pb^2+^ ions. For the pDOS of I in Figure 1f, the coordination of Fc_2_Tc_2_ elevates the valence band maximum (VBM) of the perovskite by 0.2 eV relative to both the bulk and the surface, which stems from the extra I-5p electrons after forming a hybrid complex [PbI_4_–Fc_2_Tc_2_]^3–^, revealing the active carriers generated on the perovskite surface due to the coordination effect of Fc_2_Tc_2_. The detailed electronic structure analysis is presented in Note S4 and Figures S25–S27.

### Defect Recombination and Carrier Dynamics Studies

**Figure 2:**
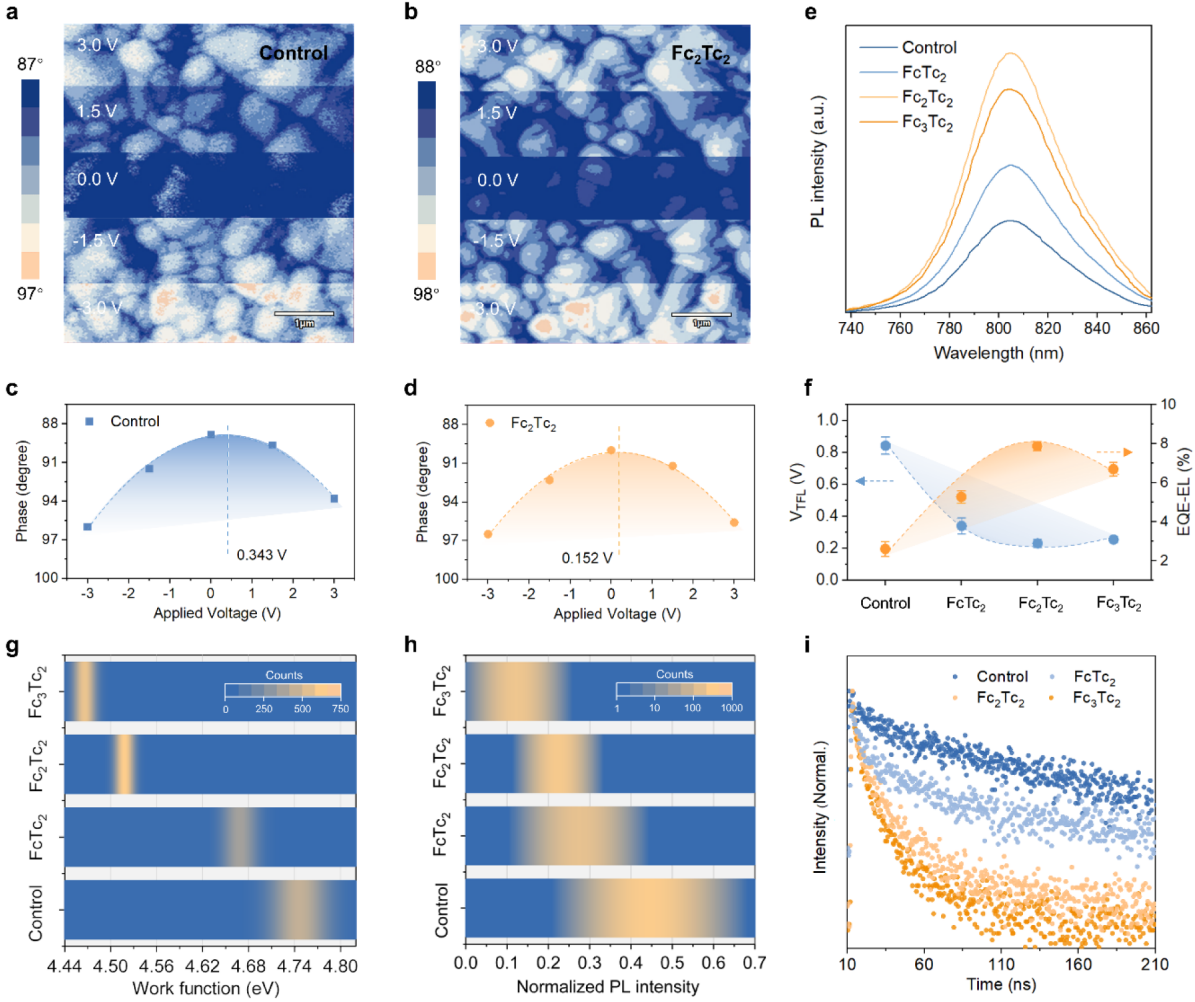
Potential evolution and carrier dynamics. (a) and (b) EFM phase images of the perovskite films with and without Fc_2_Tc_2_, where the bias voltage is supplied to the tip (−3 to 3 V, 1.5 V step) to enable the extraction of Coulombic forces. (c) and (d) EFM phase plots of the perovskite films with and without Fc_2_Tc_2_ in relation to applied bias. (e) Steady-state PL spectra of the perovskite films with different Fc compounds. The incident excitation light enters from the film side. (f) Statistics of the trap-filling voltage *V*_TFL_ and the EQE-EL values for perovskite devices. (g) Surface potential statistical maps of the perovskite films with different Fc compounds. (h) PL mapping intensity statistics for perovskite/ETL films with different Fc compounds. (i) TRPL spectra of perovskite/ETL films with different Fc compounds. The incident excitation light entered from the ETL side.

To visualize the evolution of the surface charge under the interaction between the poly ferrocenyl species and the perovskite, we performed electrostatic force microscopy (EFM) on perovskite thin films.^39,40^ The control and Fc_2_Tc_2_-treated films are displayed in Figure 2a,b and show integrated phase shift mappings throughout the whole scan region at various bias voltages, with corresponding phase angle statistics shown in Figures S28 and S29. In Figure 2c, d, the negative shift of the fitted parabola symmetry axis corresponds to the negative charges produced at the sample surface.^41^ For the Fc_2_Tc_2_-treated film, the number of negative charges rises, indicating that electrons are transferred from Fc_2_Tc_2_ to the perovskite surface, which confirms electron activation due to the formation of a resonant hybrid. Moreover, we attribute the charge change to be via the surface layer of the perovskite film, since the Fc-derived compounds are only bound to the perovskite surface according to the time-of-flight secondary ion mass spectrometry (TOF-SIMS) in Figure S30.

We used steady-state photoluminescence (PL), space-charge-limited-current (SCLC), and electroluminescence (EL) to estimate the interfacial trap states.^42^ From Figure 2e, the Fc_2_Tc_2_-treated perovskite films show higher PL intensity than the control and FcTc_2_-treated films. From Figure 2f and Figures S31–S33, after modification by the ferrocenyl compounds, the average trap-filling voltage (*V*_TFL_) decreases from 0.84 V (control) to 0.23 V (Fc_2_Tc_2_). Moreover, the average EL value increased from 2.6% (control) to 7.9% (Fc_2_Tc_2_). These results prove that the inclusion of Fc_2_Tc_2_ more effectively inhibits interface defects and nonradiative recombination.^43^ However, the addition of Fc_3_Tc_2_ leads to an increased *V*_TFL_ and a decreased EL, suggesting this effect is diminished when more Fc units are included. This increased defect density of Fc_3_Tc_2_ over Fc_2_Tc_2_ can be explained by cyclic voltammetry and molecular structure simulations (Notes S1 and S2). One consideration is the difficulty of the central Fc moiety to reduce once oxidized, with Fc_3_Tc_2_ becoming trapped in the +3 oxidation state. Additionally, the more disordered conformation of the Fc_3_Tc_2_ compound resulting from excess Fc units may act as an electron trap and cause nonradiative recombination at the perovskite-C_60_ interface.

Kelvin probe force microscopy (KPFM) was used to measure the potential evolution of the perovskite surface, and the corresponding work functions were obtained from the contact potential difference (CPD) images in Figure S34.^44^ In the surface potential color maps of Figure 2g, the control film produces an average work function of 4.746 eV with a standard deviation (SD) of 0.0264. In contrast, the ferrocenyl treatment lowers the work function, and significantly improves the surface potential uniformity, with a minimum SD value of 0.0085 for the Fc_2_Tc_2_-treated perovskite film, which enables the reduction of nonradiative recombination losses and the homogenization of charge extraction.^45^

For validating how the surface activation affects charge extraction, PL mapping was performed on the light absorber/electron transport layer (ETL) with a structure of glass/perovskite/C_60_ (Figure S35). The statistical maps of PL intensity are shown in Figure 2h, where the *x*-axis is PL intensity, and the color band represents PL intensity homogeneity. The control film shows a wide PL intensity distribution, indicating an imbalance in charge extraction. By contrast, the introduction of the ferrocenyl compounds leads to a reduced PL intensity, and the ferrocenyl-treated films display more uniform PL emission, which further verifies that the carrier extraction is improved and homogenized.^46^ In Figure 2i, the carrier lifetime, obtained by fitting the time-resolved PL spectra (Table S6), decreases from 102.44 to 4.87 ns as the number of Fc units increases. The reduction of PL intensity and lifetime implies improved charge extraction from the perovskite to ETL.^11^ Although Fc_3_Tc_2_-treated films exhibit the most pronounced PL quenching, part of this quenching comes from defect-induced nonradiative recombination due to the increased trap states in the Fc_3_Tc_2_-treated films. For these reasons and the relative ease of synthesis, Fc_2_Tc_2_ was focused for in-depth evaluation of photovoltaic performance.

### Photovoltaic Performance of Small- and Large-Area Devices

**Figure 3:**
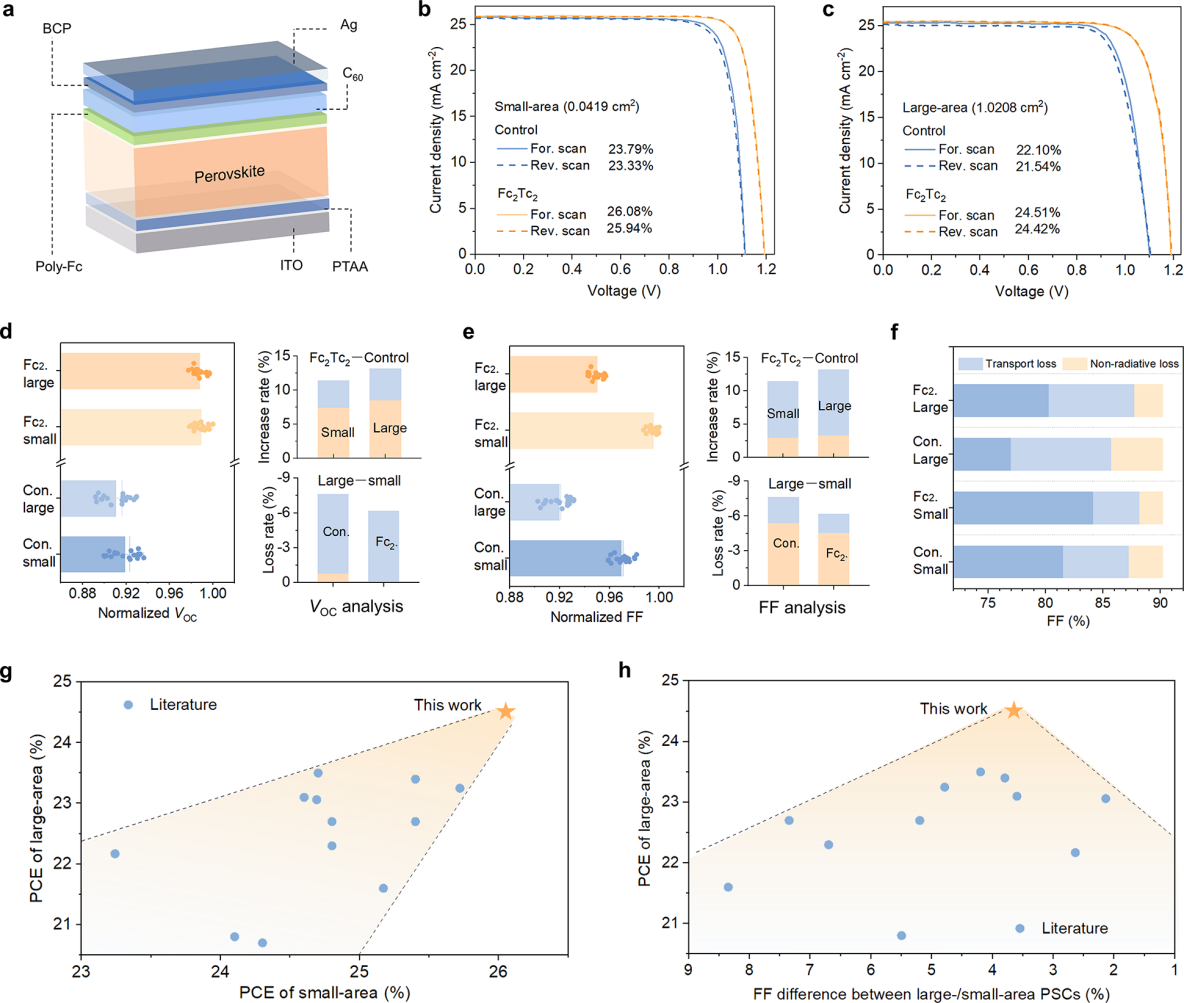
Photovoltaic performance. (a) Schematic illustration of the PSC device structure. (b) *J*–*V* curves of the best-performing small-area PSCs with and without Fc_2_Tc_2_. (c) *J*–*V* curves of the best-performing large-area PSCs with and without Fc_2_Tc_2_. (d) and (e) Statistics of normalized *V*_OC_ (d) and FF (e) for 20 individual small- and large-area devices with and without Fc_2_Tc_2_. The scatter points represent the parameters of each device, and the histogram is the average value. Data are normalized to the highest value of each parameter. (f) FF loss calculation in small- and large-area devices with and without Fc_2_Tc_2_. (g) and (h) Recent literature statistics (Table S3) on champion PCEs for the simultaneous preparation of small- and large-area PSCs.

The photovoltaic performance was studied on a device configuration consisting of indium tin oxide (ITO)/poly[bis(4-phenyl) (2,4,6-trimethylphenyl) amine] (PTAA)/perovskite/Fc-derived molecules/C_60_/2,9-dimethyl-4,7-diphenyl-1,10 phenanthroline (BCP)/ silver (Ag), as illustrated in Figures 3a and S36. Figure 3b shows the current–voltage (*J*–*V*) curves of the small-area PSCs with and without Fc_2_Tc_2_. The control device exhibits a maximum PCE of 23.79%, with a *V*_OC_ of 1.113 V, a short-circuit current density (*J*_SC_) of 25.82 mA cm^–2^, and an FF of 82.78%. The introduction of ferrocenyl molecules induces a significant increase in PCEs as shown in Table S7 and Figure S37. The Fc_2_Tc_2_-modified device produces a champion PCE of 26.08%, with a *V*_OC_ of 1.194 V, a *J*_SC_ of 25.93 mA cm^–2^, and a FF of 84.24%. In addition, the performance of Fc_2_Tc_2_-modified devices at various concentrations is summarized in Table S8 and Figure S38. FcTc_2_- and Fc_3_Tc_2_-modified devices display similar performances to each other with maximum PCEs of 25.43 and 25.82%, respectively (Figure S37).

After demonstrating the exceptional performance in small-area devices, we proceeded to fabricate large-area cells (with an active area of 1.0208 cm^2^), focusing on the use of Fc_2_Tc_2_. As shown in Figure 3c, the control device demonstrates a *J*_SC_ of 25.30 mA cm^–2^, a *V*_OC_ of 1.110 V, and a FF of 78.68%, which produces a PCE of 22.10%. The Fc_2_Tc_2_-treated devices exhibit a similar performance trend to those of small-area devices (Table S9 and Figure S39). Strikingly, the Fc_2_Tc_2_-treated device shows significant improvements in FF (80.82%) and *V*_OC_ (1.190 V), ultimately achieving a champion PCE of 24.51%, which is the highest value among all large-area PSCs of similar size. It is worth noting that the Fc_2_Tc_2_-based devices display negligible hysteresis for both large- and small-areas, which is much smaller than those of the control devices. One of the best-performing small-area and large-area PSCs was certified by an independent solar cell certification laboratory (SIMIT, China), where a PCE of 25.83% (*V*_OC_ = 1.191 V, *J*_SC_ = 25.86 mA cm^–2^, and FF = 83.88%) for the small-area device and a PCE of 23.77% (*V*_OC_ = 1.184 V, *J*_SC_ = 25.37 mA cm^–2^, and FF = 79.16%) for the large-area device were confirmed (See Figures S40 and S41).

To elucidate the origin of the performance enhancement, we conducted statistical analyses on the photovoltaic parameters of 20 individual devices in Figure S42. The PCE improvements for both small- and large-area devices are mainly attributed to the increased *V*_OC_ and FF after incorporating Fc_2_Tc_2_, as shown in Figure 3d and 3e. Notably, the *V*_OC_ increment contributes more significantly to the PCE augmentation for both small- and large-area devices, with contribution rates of 65% and 64%, respectively. Moreover, in Figures S43 and S44, the quasi-Fermi level splitting (QFLS) analyses of the ITO/HTL/perovskite/ETL stack are 1.12 V vs 1.098 V (average *V*_OC_) for the control device and 1.189 V vs 1.180 V (average *V*_OC_) for the Fc_2_Tc_2_-modified device, respectively, suggesting a reduced voltage loss after Fc_2_Tc_2_ treatment.^47^

For FF, we observed that although FF contributes 25% to the performance boost for both large- and small-area devices after modification, the FF loss is the primary origin of the efficiency discrepancy between large- and small-area devices. While the introduction of Fc_2_Tc_2_ mitigates the FF loss from 5.4% to 4.5% (Figure 3e), eventually resulting in a reduction of efficiency loss from 7.6% to 6.1%. We further calculated the FF losses through the ideality factor (Figure S45).^48^ In Figure 3f, it is evident that the small-area devices with inclusion of Fc_2_Tc_2_ exhibit substantially lower nonradiative recombination loss and transport loss than the control devices. The same trend is also observed in large-area devices. This clearly indicates that Fc_2_Tc_2_ can effectively suppress nonradiative recombination and lower the resistance barrier for interfacial transport.

The stable power output (SPO) curves of large-area devices (Figure S46) demonstrate stable device operation at maximum power point (MPP). The external quantum efficiency (EQE) spectrum of the large-area device is presented in Figure S47, showing similar integrated current for the control and Fc_2_Tc_2_-modified devices. It is noteworthy that, the suppression of recombination and transport loss collectively enhance the performance of large/small area PSCs and narrow the FF and PCE gap between them, leading to a greater improvement in their efficiency, as depicted in Figure 3g,h and Table S3.

### Photovoltaic Uniformity and Stability

**Figure 4:**
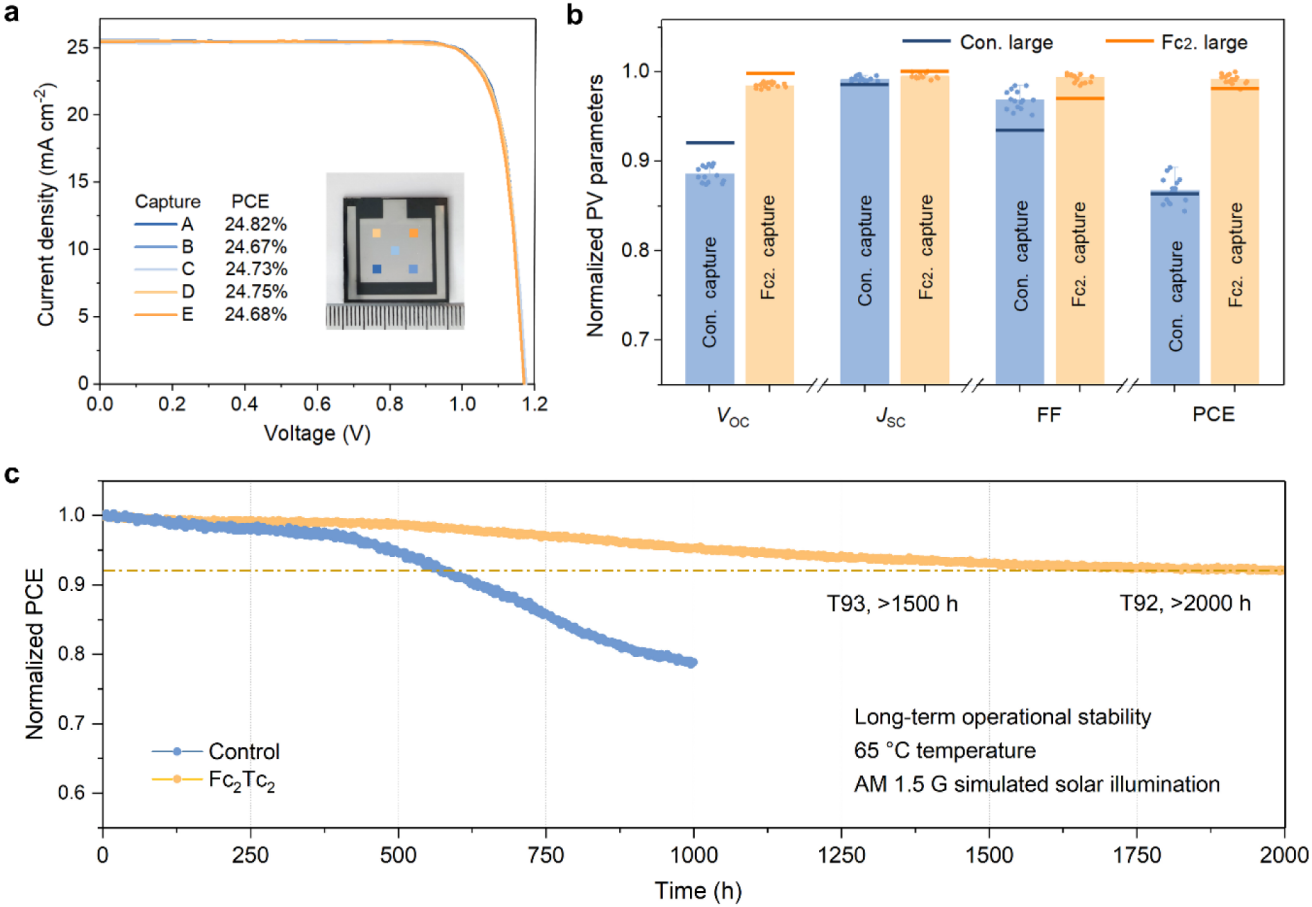
Large-area photovoltaic uniformity and stability. (a) *J*–*V* curves of a representative Fc_2_Tc_2_-treated large-area PSC measured from five different spots captured using a small-area mask. (b) Statistics of the normalized photovoltaic parameters of the small-area PSCs captured from large-area PSCs. The data were recorded from five separate points on three devices and normalized to the highest value of each parameter. The lines represent the average photovoltaic parameters for large-area devices. (c) Normalized PCE evolution of the encapsulated large-area devices measured at the MPP under continuous AM 1.5 G simulated solar illumination at 65 °C under N_2_.

For further assessment of the photovoltaic performance homogeneity of large-area devices, we recorded *J*–*V* curves of the devices captured at five separate places, i.e., positioned in the center and four corners of the device active region (Figure 4a). All of the photovoltaic metrics of the Fc_2_Tc_2_-treated device, obtained from the *J*–*V* curves at these five places using a small-area mask, show minimal variation. Upon expanding the volume of statistics as shown in Figure 4b, the *V*_OC_ of the captured small-area devices is lower than that of large-area devices, while the trend is the opposite for FF. Moreover, Fc_2_Tc_2_ treatment enables more uniform performance in different locations. Significantly, the Fc_2_Tc_2_-treated devices exhibit narrower performance differences between the large-area and captured small-area devices (mainly for *V*_OC_ and FF). We used KPFM to measure the surface potential of different regions in large-area perovskite films. Figures S48 and S49 show that the ferrocenyl modification makes the surface potential more uniform at each individual region. Although the potentials at different locations deviate slightly, Fc_2_Tc_2_ reduces nonradiative recombination for each localized location, enabling optimal carrier extraction for large-area perovskite device, and boosting the performance of scalable PSCs.

The long-term operating stabilities were studied by examining the encapsulated large-area devices at the MPP at 65 °C under continuous one-sun illumination under a N_2_ atmosphere (following the ISOS-L-2I protocol).^49^ The initial efficiencies of the devices before testing were 21.66 and 23.95%, respectively, which are both at the average level for the control and the Fc_2_Tc_2_ devices, respectively (Figure S50). After testing, the Fc_2_Tc_2_-treated device demonstrates outstanding stability, with over 93% PCE (>T93) after 1500 h and 92% PCE (>T92) after 2000 h as shown in Figure 4c. By contrast, the control device can retain similar stability variation over 300 h, while losing more than 20% of its initial PCE after 1000 h.

## Conclusions

In summary, we have demonstrated a facile and effective approach to fabricate p-i-n PSCs with an impressive PCE of 26.08% for small-area devices and 24.51% for large-area devices through surface manipulation using the bioferrocenyl compound Fc_2_Tc_2_. In-depth analysis of molecular interactions, electronic structure, carrier dynamics, and photovoltaic performance provide insights into the role of ferrocene-induced resonant hybrids in reducing nonradiative recombination and charge transport losses, ultimately in increased *V*_OC_ and reduced FF loss as expanding PSC area. This breakthrough in enhancing the PCEs of both small- and large-area devices demonstrates the potential of ferrocenyl molecules, opening up a new avenue for developing commercially viable, high-performance, and scalable p-i-n PSCs.

## Data Availability

All data needed to evaluate the conclusions in the paper are present in the paper or the supporting materials.

## Supporting Information Available

Description of experimental details, materials and methods, additional simulations details and characterization data, including NMR, cyclic voltammetry, UV–vis. absorption spectra, X-ray diffraction and structure analyses, XPS, SEM, AFM, KPFM, TOF-SIMS, SCLC, PL mapping, EQE and EL curves, *J*–*V* curves, and independent efficiency certification reports (PDF)

## References

[ref1] Luo D.; Yang W.; Wang Z.; Sadhanala A.; Hu Q.; Su R.; Shivanna R.; Trindade G. F.; Watts J. F.; Xu Z.; et al. Enhanced photovoltage for inverted planar heterojunction perovskite solar cells. Science 2018, 360, 1442–1446. 10.1126/science.aap9282. 29954975

[ref2] Chen H.; Teale S.; Chen B.; Hou Y.; Grater L.; Zhu T.; Bertens K.; Park S. M.; Atapattu H. R.; Gao Y.; et al. Quantum-size-tuned heterostructures enable efficient and stable inverted perovskite solar cells. Nat. Photonics 2022, 16, 352–358. 10.1038/s41566-022-00985-1.

[ref3] Jiang Q.; Tong J. H.; Xian Y. M.; Kerner R. A.; Dunfield S. P.; Xiao C. X.; Scheidt R. A.; Kuciauskas D.; Wang X. M.; Hautzinger M. P.; et al. Surface reaction for efficient and stable inverted perovskite solar cells. Nature 2022, 611, 278–283. 10.1038/s41586-022-05268-x. 36049505

[ref4] Min H.; Lee D. Y.; Kim J.; Kim G.; Lee K. S.; Kim J.; Paik M. J.; Kim Y. K.; Kim K. S.; Kim M. G.; et al. Perovskite solar cells with atomically coherent interlayers on SnO_2_ electrodes. Nature 2021, 598, 444–450. 10.1038/s41586-021-03964-8. 34671136

[ref5] Zheng X.; Hou Y.; Bao C.; Yin J.; Yuan F.; Huang Z.; Song K.; Liu J.; Troughton J.; Gasparini N.; et al. Managing grains and interfaces via ligand anchoring enables 22.3%-efficiency inverted perovskite solar cells. Nat. Energy 2020, 5, 131–140. 10.1038/s41560-019-0538-4.

[ref6] Cao Q.; Li Y.; Zhang H.; Yang J.; Han J.; Xu T.; Wang Z. S.; Wang Z.; Gao B.; Zhao J. Efficient and stable inverted perovskite solar cells with very high fill factors via incorporation of star-shaped polymer. Sci. Adv. 2021, 7 (28), eabg0633 10.1126/sciadv.abg0633. 34233877 PMC8262814

[ref7] Li Z.; Li B.; Wu X.; Sheppard S. A.; Zhang S.; Gao D.; Long N. J.; Zhu Z. Organometallic-functionalized interfaces for highly efficient inverted perovskite solar cells. Science 2022, 376, 416–420. 10.1126/science.abm8566. 35446656

[ref8] Degani M.; An Q.; Albaladejo-Siguan M.; Hofstetter Y. J.; Cho C.; Paulus F.; Grancini G.; Vaynzof Y. 23.7% Efficient inverted perovskite solar cells by dual interfacial modification. Sci. Adv. 2021, 7 (49), eabj7930 10.1126/sciadv.abj7930. 34851671 PMC8635431

[ref9] Li X.; Zhang W.; Guo X.; Lu C.; Wei J.; Fang J. Constructing heterojunctions by surface sulfidation for efficient inverted perovskite solar cells. Science 2022, 375, 434–437. 10.1126/science.abl5676. 35084976

[ref10] Azmi R.; Ugur E.; Seitkhan A.; Aljamaan F.; Subbiah A. S.; Liu J.; Harrison G. T.; Nugraha M. I.; Eswaran M. K.; Babics M.; et al. Damp heat–stable perovskite solar cells with tailored-dimensionality 2D/3D heterojunctions. Science 2022, 376, 73–77. 10.1126/science.abm5784. 35175829

[ref11] Rong Y.; Hu Y.; Mei A.; Tan H.; Saidaminov M. I.; Seok S. I.; McGehee M. D.; Sargent E. H.; Han H. Challenges for commercializing perovskite solar cells. Science 2018, 361 (6408), eaat8235 10.1126/science.aat8235. 30237326

[ref12] Chen S.; Dai X.; Xu S.; Jiao H.; Zhao L.; Huang J. Stabilizing perovskite-substrate interfaces for high-performance perovskite modules. Science 2021, 373, 902–907. 10.1126/science.abi6323. 34413234

[ref13] Du M.; Zhao S.; Duan L.; Cao Y.; Wang H.; Sun Y.; Wang L.; Zhu X.; Feng J.; Liu L.; et al. Surface redox engineering of vacuum-deposited NiO_x_ for top-performance perovskite solar cells and modules. Joule 2022, 6, 1931–1943. 10.1016/j.joule.2022.06.026.

[ref14] Wu Y.; Yang X.; Chen W.; Yue Y.; Cai M.; Xie F.; Bi E.; Islam A.; Han L. Perovskite solar cells with 18.21% efficiency and area over 1 cm^2^ fabricated by heterojunction engineering. Nat. Energy 2016, 1 (11), 16148 10.1038/nenergy.2016.148.

[ref15] Yang J.; Cao Q.; Wang T.; Yang B.; Pu X.; Zhang Y.; Chen H.; Tojiboyev I.; Li Y.; Etgar L.; et al. Inhibiting metal-inward diffusion-induced degradation through strong chemical coordination toward stable and efficient inverted perovskite solar cells. Energy Environ. Sci. 2022, 15, 2154–2163. 10.1039/D1EE04022G.

[ref16] Li Z.; Klein T. R.; Kim D. H.; Yang M.; Berry J. J.; van Hest M. F. A. M.; Zhu K. Scalable fabrication of perovskite solar cells. Nat. Rev. Mater. 2018, 3 (4), 18017 10.1038/natrevmats.2018.17.

[ref17] Chen W.; Wu Y.; Yue Y.; Liu J.; Zhang W.; Yang X.; Chen H.; Bi E.; Ashraful I.; Grätzel M.; et al. Efficient and stable large-area perovskite solar cells with inorganic charge extraction layers. Science 2015, 350, 944–948. 10.1126/science.aad1015. 26516198

[ref18] Liu Z.; Qiu L.; Ono L. K.; He S.; Hu Z.; Jiang M.; Tong G.; Wu Z.; Jiang Y.; Son D.-Y.; et al. A holistic approach to interface stabilization for efficient perovskite solar modules with over 2,000-h operational stability. Nat. Energy 2020, 5, 596–604. 10.1038/s41560-020-0653-2.

[ref19] Peng J.; Kremer F.; Walter D.; Wu Y.; Ji Y.; Xiang J.; Liu W.; Duong T.; Shen H.; Lu T.; Brink F.; Zhong D.; Li L.; Lem O. L. C.; Liu Y.; Weber K. J.; White T. P.; Catchpole K. R. Centimetre-scale perovskite solar cells with fill factors of more than 86 per cent. Nature 2022, 601, 573–578. 10.1038/s41586-021-04216-5. 35082415

[ref20] Yoo J. J.; Seo G.; Chua M. R.; Park T. G.; Lu Y.; Rotermund F.; Kim Y. K.; Moon C. S.; Jeon N. J.; Correa-Baena J. P.; et al. Efficient perovskite solar cells via improved carrier management. Nature 2021, 590, 587–593. 10.1038/s41586-021-03285-w. 33627807

[ref21] Luo D.; Su R.; Zhang W.; Gong Q.; Zhu R. Minimizing non-radiative recombination losses in perovskite solar cells. Nat. Rev. Mater. 2020, 5, 44–60. 10.1038/s41578-019-0151-y.

[ref22] Zhang W.; Pathak S.; Sakai N.; Stergiopoulos T.; Nayak P. K.; Noel N. K.; Haghighirad A. A.; Burlakov V. M.; deQuilettes D. W.; Sadhanala A.; et al. Enhanced optoelectronic quality of perovskite thin films with hypophosphorous acid for planar heterojunction solar cells. Nat. Commun. 2015, 6, 10030 10.1038/ncomms10030. 26615763 PMC4674686

[ref23] Zheng X.; Chen B.; Dai J.; Fang Y.; Bai Y.; Lin Y.; Wei H.; Zeng X. C.; Huang J. Defect passivation in hybrid perovskite solar cells using quaternary ammonium halide anions and cations. Nat. Energy 2017, 2, 17102 10.1038/nenergy.2017.102.

[ref24] Sherkar T. S.; Momblona C.; Gil-Escrig L.; Bolink H. J.; Koster L. J. A. Improving perovskite solar cells: Insights from a validated device model. Adv. Energy Mater. 2017, 7, 1602432 10.1002/aenm.201602432.

[ref25] Stolterfoht M.; Wolff C. M.; Amir Y.; Paulke A.; Perdigón-Toro L.; Caprioglio P.; Neher D. Approaching the fill factor Shockley–Queisser limit in stable, dopant-free triple cation perovskite solar cells. Energy Environ. Sci. 2017, 10, 1530–1539. 10.1039/C7EE00899F.

[ref26] Mosa I. M.; Pattammattel A.; Kadimisetty K.; Pande P.; El-Kady M. F.; Bishop G. W.; Novak M.; Kaner R. B.; Basu A. K.; Kumar C. V.; et al. Ultrathin Graphene-Protein Supercapacitors for Miniaturized Bioelectronics. Adv. Energy Mater. 2017, 7 (17), 1702358 10.1002/aenm.201700358. PMC5667682 29104523

[ref27] Li B.; Li S.; Gong J.; Wu X.; Li Z.; Gao D.; Zhao D.; Zhang C.; Wang Y.; Zhu Z. Fundamental understanding of stability for halide perovskite photovoltaics: The importance of interfaces. Chem 2024, 10, 35–47. 10.1016/j.chempr.2023.09.002.

[ref28] Lin Q.; Nagiri R. C. R.; Burn P. L.; Meredith P. Considerations for upscaling of organohalide perovskite solar cells. Adv. Opt. Mater. 2017, 5, 1600819 10.1002/adom.201600819.

[ref29] Kresse G. Ab-Initio Molecular-Dynamics for Liquid-Metals. J. Non-Cryst. Solids 1995, 192, 222–229. 10.1016/0022-3093(95)00355-X.

[ref30] Blöchl P. E. Projector Augmented-Wave Method. Phys. Rev. B 1994, 50, 17953–17979. 10.1103/PhysRevB.50.17953. 9976227

[ref31] Perdew J. P.; Burke K.; Ernzerhof M. Generalized Gradient Approximation Made Simple. Phys. Rev. Lett. 1996, 77, 3865–3868. 10.1103/PhysRevLett.77.3865. 10062328

[ref32] Grimme S. Semiempirical GGA-type density functional constructed with a long-range dispersion correction. J. Comput. Chem. 2006, 27, 1787–1799. 10.1002/jcc.20495. 16955487

[ref33] Grimme S.; Antony J.; Ehrlich S.; Krieg H. A consistent and accurate ab initio parametrization of density functional dispersion correction (DFT-D) for the 94 elements H-Pu. J. Chem. Phys. 2010, 132, 154104 10.1063/1.3382344. 20423165

[ref34] Moellmann J.; Grimme S. DFT-D3 Study of Some Molecular Crystals. J. Phys. Chem. C 2014, 118 (14), 7615–7621. 10.1021/jp501237c.

[ref35] Oner S. M.; Sezen E.; Yordanli M. S.; Karakoc E.; Deger C.; Yavuz I. Surface defect formation and passivation in formamidinium lead triiodide (FAPbI_3_) perovskite solar cell absorbers. J. Phys. Chem. Lett. 2022, 13, 324–330. 10.1021/acs.jpclett.1c03645. 34978837

[ref36] Park K.; Lee J. H.; Lee J. W. Surface defect engineering of metal halide perovskites for photovoltaic applications. ACS Energy Lett. 2022, 7, 1230–1239. 10.1021/acsenergylett.1c02847.

[ref37] Gao Y.; Raza H.; Zhang Z.; Chen W.; Liu Z. Rethinking the role of excess/residual lead iodide in perovskite solar cells. Adv. Funct. Mater. 2023, 33, 2215171 10.1002/adfm.202215171.

[ref38] Haruyama J.; Sodeyama K.; Han L.; Tateyama Y. Termination dependence of tetragonal CH_3_NH_3_PbI_3_ surfaces for perovskite solar cells. J. Phys. Chem. Lett. 2014, 5, 2903–2909. 10.1021/jz501510v. 26278097

[ref39] Mélin T.; Diesinger H.; Deresmes D.; Stiévenard D. Electric force microscopy of individually charged nanoparticles on conductors: an analytical model for quantitative charge imaging. Phys. Rev. B 2004, 69, 035321 10.1103/PhysRevB.69.035321.

[ref40] Heim T.; Lmimouni K.; Vuillaume D. Ambipolar charge injection and transport in a single pentacene monolayer island. Nano Lett. 2004, 4, 2145–2150. 10.1021/nl0487673.

[ref41] Chen W.; Zhou Y.; Wang L.; Wu Y.; Tu B.; Yu B.; Liu F.; Tam H.-W.; Wang G.; Djurǐić A. B.; et al. Molecule-doped nickel oxide: Verified charge transfer and planar inverted mixed cation perovskite solar cell. Adv. Mater. 2018, 30, 1800515 10.1002/adma.201800515. 29603421

[ref42] Li B.; Wu X.; Zhang H.; Zhang S.; Li Z.; Gao D.; Zhang C.; Chen M.; Xiao S.; Jen A. K. Y.; et al. Efficient and Stable Tin Perovskite Solar Cells by Pyridine-Functionalized Fullerene with Reduced Interfacial Energy Loss. Adv. Funct. Mater. 2022, 32, 2205870 10.1002/adfm.202205870.

[ref43] Jiang Y. T.; Wang J. B.; Zai H. C.; Ni D. Y.; Wang J. Y.; Xue P. Y.; Li N. X.; Jia B. Y.; Lu H. J.; Zhang Y.; et al. Reducing energy disorder in perovskite solar cells by chelation. J. Am. Chem. Soc. 2022, 144, 5400–5410. 10.1021/jacs.1c12732. 35306820

[ref44] Li B.; L Q.; Gong J.; Li S.; Zhang C.; Gao D.; Chen Z.; Li Z.; Wu X.; Zhao D. Harnessing strong aromatic conjugation in low-dimensional perovskite heterojunctions for high-performance photovoltaic devices. Nat. Commun. 2024, 15, 2753 10.1038/s41467-024-47112-y. 38553436 PMC10980693

[ref45] Li N.; Niu X.; Li L.; Wang H.; Huang Z.; Zhang Y.; Chen Y.; Zhang X.; Zhu C.; Zai H.; et al. Liquid medium annealing for fabricating durable perovskite solar cells with improved reproducibility. Science 2021, 373, 561–567. 10.1126/science.abh3884. 34326239

[ref46] Jiang X.; Liu B.; Wu X.; Zhang S.; Zhang D.; Wang X.; Gao S.; Huang Z.; Wang H.; Li B.; Top-down Induced Crystallization Orientation toward Highly Efficient p-i-n Perovskite Solar Cells, Adv. Mater., 2024, 2313524. 10.1002/adma.202313524. 38453665

[ref47] Li Z.; Sun X.; Zheng X.; Li B.; Gao D.; Zhang S.; Wu X.; Li S.; Gong J.; Luther J. M.; et al. Stabilized hole-selective layer for high-performance inverted pin perovskite solar cells. Science 2023, 382, 284–289. 10.1126/science.ade9637. 37856581

[ref48] Tian J.; Zhang K.; Xie Z.; Peng Z.; Zhang J.; Osvet A.; Lüer L.; Kirchartz T.; Rau U.; Li N.; Brabec C. J. Quantifying the Energy Losses in CsPbI_2_Br Perovskite Solar Cells with an Open-Circuit Voltage of up to 1.45 V. ACS Energy Lett. 2022, 7 (11), 4071–4080. 10.1021/acsenergylett.2c01883.

[ref49] Khenkin M. V.; Katz E. A.; Abate A.; Bardizza G.; Berry J. J.; Brabec C.; Brunetti F.; Bulović V.; Burlingame Q.; Di Carlo A.; et al. Consensus statement for stability assessment and reporting for perovskite photovoltaics based on ISOS procedures. Nat. Energy 2020, 5, 35–49. 10.1038/s41560-019-0529-5.

