# Supplementary material for: Highly Efficient and Scalable p-i-n Perovskite Solar Cells Enabled by Poly-metallocene Interfaces
Source: J Am Chem Soc. 2024 May 1;146(19):13391–8. doi: 10.1021/jacs.4c02220 (PMC11100013; doi:10.1021/jacs.4c02220)
Supplement: Supplementary file 1 — ja4c02220_si_001.pdf [file ja4c02220_si_001.pdf]

Supporting Information

Highly efficient and scalable p-i-n perovskite solar cells enabled by poly-metallocene interfaces

Bo Li[1,#], Danpeng Gao[1,#], Stephanie A. Sheppard[2,#], William D. J. Tremlett[2,#], Qi Liu[3,#], Zhen Li[1], Andrew J. P. White[2], Ryan K. Bown[2], Xianglang Sun[1], Jianqiu Gong[1], Shuai Li[1], Shoufeng Zhang[1], Xin Wu[1], Dan Zhao[1], Chunlei Zhang[1], Yan Wang[1], Xiao Cheng Zeng[3*], Zonglong Zhu[1*] and Nicholas J Long[2*]

[1] Department of Chemistry, City University of Hong Kong, Kowloon 999077, Hong Kong

[2] Department of Chemistry, Imperial College London, MSRH Building, White City Campus, London, W12 0BZ, UK

[3] Department of Materials Science & Engineering, City University of Hong Kong, Kowloon 999077, Hong Kong

* Corresponding author's email: n.long@imperial.ac.uk; zonglzhu@cityu.edu.hk; xzeng26@cityu.edu.hk

# These authors contributed equally

Keywords: p-i-n perovskite solar cells, poly-ferrocene compounds, high efficiency, scalability

Materials and Methods

Materials

Formamidinium iodide (FAI), methylammonium bromide (MABr), methylammonium chloride (MACl) and cesium iodide (CsI) were purchased from Dysol (Australia). Lead iodide (PbI_2) and lead bromide ($PbBr_2$) were purchased from TCI (Japan). C_{60}, poly[bis(4-phenyl)(2,4,6-trimethylphenyl) amine] (PTAA) (*M*n 6,000-15,000), and bathocuproine (BCP, 99.9%) were purchased from Xi'an Polymer Light Technology Corporation (China). High purity silver was purchased from commercial sources. Glass substrates patterned with indium tin oxide (ITO) (15 Ω sq^{-1}) were received from Mishi Tech. Co., Ltd. (China). Dimethylformamide (DMF), dimethyl sulfoxide (DMSO), isopropanol (IPA) and chlorobenzene (CB) were purchased from J&K (China) and used as received. Dichloromethane (CH_2Cl_2) and *n*-hexane were purchased from VWR Chemicals, and deuterated chloroform ($CDCl_3$) was purchased from Sigma-Aldrich. Acetonitrile (CH_3CN) was dried through a solvent purification system under a nitrogen atmosphere (Inert PureSolv) and transferred into Schlenk flasks that were dried under vacuum and purged with N_2 prior to use. Copper(I) oxide (Acros Organics, 97%) and thiophene-2-carboxylic acid (Alfa Aesar, 99%) were used without further purification. Diiodobiferrocene (Fc_2I_2),[1] diiodotriferrocene (Fc_3I_2)[1] and ferrocenyl-bis-thiophene-2-carboxylate ($FcTc_2$)[2] were prepared using modified literature procedures.

General procedure for the synthesis of biferrocenyl-bis-thiophene-2-carboxylate (Fc_2Tc_2) and triferrocenyl-bis-thiophene-2-carboxylate (Fc_3Tc_2)

Fc_2I_2 or Fc_3I_2 (1 equiv), Cu_2O (1.5 equiv) and thiophene-2-carboxylic acid (3 equiv) were refluxed for 24 h in CH_3CN. The rection mixture was diluted with CH_2Cl_2, filtered and the filtrate washed with saturated aqueous $NaHCO_3$ until the aqueous phase became colorless. The organic phase was dried over Na_2SO_4, filtered and concentrated *in vacuo*. The crude residue was purified by flash chromatography (*n*-hexane/CH_2Cl_2) and the resulting product recrystallized from CH_2Cl_2 and *n*-hexane to afford the desired compound.

Biferrocenyl-bis-thiophene-2-carboxylate (Fc_2Tc_2)

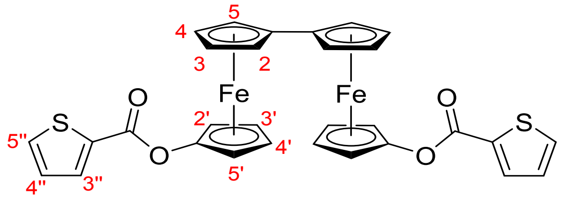

Fc_2Tc_2 was prepared according to the general procedure using Fc_2I_2 (947 mg, 1.52 mmol), 2-thiophenecarboxylic acid (585 mg, 4.57 mmol), Cu_2O (327 mg, 2.28 mmol) and CH_3CN (80 mL) to afford orange crystals (652 mg, 69%). R_f 0.32 (CH_2Cl_2/*n*-hexane 1:1); ^{1}H NMR (400 MHz, $CDCl_3$): δ 7.76 (dd, 2H, ^3J = 3.8, ^4J = 1.2 Hz, 2 × H-5''), 7.59 (dd, 2H, ^3J = 4.9, ^4J = 1.3 Hz, 2 × H-3''), 7.11 (dd, 2H, ^3J = 5.0, ^3J = 3.7 Hz, 2 × H-4''), 4.44 (pseudo-t, 4H, J = 1.8 Hz, 2 × H-3 and H-4), 4.39 (pseudo-t, 4H, J = 2.0 Hz, 2 × H-3' and H-4'), 4.16 (pseudo-t, 4H, J = 1.8 Hz, 2 × H-2 and H-5), 3.81 (pseudo-t, 4H, J = 1.9 Hz, 2 × H-2' and H-5'); ^{13}C{^{1}H} NMR (100 MHz, $CDCl_3$): δ 160.4 (2 × C, 2 × C=O), 134.2 (2 × CH, 2 × C-5''), 133.4 (2 × C, 2 × C-2''), 133.0 (2 × CH, 2 × C-3''), 127.9 (2 × CH, 2 × C-4''), 116.3 (2 × C, 2 × 1'), 84.4 (2 × C, 2 × C-1), 69.2 (4 × CH, 2 × C-2 and C-5), 67.7 (4 × CH, 2 × C-3 and C-4), 64.5 (4 × CH, 2 × C-2' and C-5'), 62.0 (4 × CH, 2 × C-3' and C-4'); HRMS (ESI$^+$): *m/z* 621.9655 $[M]^+$ (m_{calc} 621.9658); Calcd for $C_{30}H_{22}Fe_2O_4S_2$: C 57.90, H 3.56. Found: C 57.68, H 3.49.

Triferrocenyl-bis-thiophene-2-carboxylate (Fc_3Tc_2)

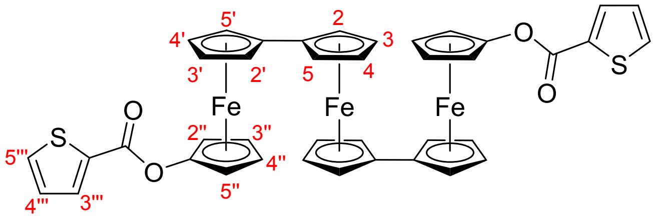

Fc_3Tc_2 was prepared according to the general procedure using Fc_3I_2 (718 mg, 0.89 mmol), 2-thiophenecarboxylic acid (343 mg, 2.67 mmol), Cu_2O (191 mg, 1.34 mmol) and CH_3CN (100 mL) to afford red crystals (0.36 mg, 50%). R_f 0.42 (CH_2Cl_2/*n*-hexane 3:2); ^{1}H NMR (400 MHz, $CDCl_3$): δ 7.73 (dd, 2H, ^3J = 3.7 Hz, ^4J = 1.3 Hz, 2 × H-5'''), 7.58 (dd, 2H, ^3J = 5.0 Hz, ^4J = 1.3 Hz, 2 × H-3'''), 7.10 (dd, 2H, ^3J = 5.0, ^3J = 3.7 Hz, 2 × H-4'''), 4.33 (pseudo-t, 4H, J = 2.0 Hz, 2 × H-3'' and H-4''), 4.30 (pseudo-t, 4H, J = 1.8 Hz, 2 × H-3 and H-4 or 2 × H-3' and H-4'), 4.21 (pseudo-t, 4H, J = 1.9 Hz, 2 × H-2 and H-5 or 2 × H-2' and H-5'), 4.06 (pseudo-t, 4H, J =

1.9 Hz, 2 × H-3 and H-4 or 2 × H-3' and H-4'), 3.89 (pseudo-t, 4H, J = 1.8 Hz, 2 × H-2 and H-5 or 2 × H-2' and H-5'), 3.77 (pseudo-t, 4H, J = 2.0 Hz, 2 × H-2'' and H-5''); $^{13}C\{^1H\}$ NMR (100 MHz, $CDCl_3$): δ 160.4 (2 × C, 2 × C=O), 134.2 (2 × CH, 2 × C-5'''), 133.5 (2 × C, 2 × C-2'''), 133.0 (2 × CH, 2 × C-3'''), 127.9 (2 × CH, 2 × C-4'''), 116.2 (2 × C, 2 × 1''), 85.1 (2 × C, 2 × C-1 or 2 × C-1'), 83.3 (2 × C, 2 × C-1 or 2 × C-1'), 69.0 (4 × CH, 2 × C-2 and C-5 or 2 × C-2' and C-5'), 69.0 (4 × CH, 2 × C-2 and C-5 or 2 × C-2' and C-5'), 67.8 (4 × CH, 2 × C-3 and C-4 or 2 × C-3' and C-4'), 67.5 (4 × CH, 2 × C-3 and C-4 or 2 × C-3' and C-4'), 64.5 (4 × CH, 2 × C-2'' and C-5''), 61.9 (4 × CH, 2 × C-3'' and C-4''); HRMS (ESI^+): *m/z* 805.9632 $[M]^+$ (m_{calc} 805.9634); Calcd for $C_{40}H_{30}Fe_3O_4S_2$: C 59.58, H 3.75. Found: C 59.28, H 3.49.

$(FA_{0.98}MA_{0.02})_{0.95}Cs_{0.05}Pb(I_{0.98}Br_{0.02})_3$-based device fabrication

ITO substrates were sequentially cleaned with detergent, deionized water, acetone and isopropyl alcohol under ultrasonication for 20 min, respectively. Then, the ITO substrates were dried and treated with oxygen plasma for 20 min. The PTAA solution was prepared with a concentration of 2.2 mg ml^{-1} in CB. The 40 μL as-prepared PTAA solution was spin-coated onto the ITO substrates at 6000 rpm for 30 s. Subsequently, the substrates were annealed at 100 °C for 10 min. The perovskite solution (1.73 M) was prepared by mixing CsI, FAI, MABr, PbI_2 and $PbBr_2$ in 1 ml mixed DMF:DMSO (5:1/v:v) solvent for a chemical formula $(FA_{0.98}MA_{0.02})_{0.95}Cs_{0.05}Pb(I_{0.98}Br_{0.02})_3$, a certain concentration of excess PbI_2 was needed to improve the device performance. Then 15.5 mol% MACl was added to the perovskite precursor solution and stirred for 2 h. 50 μL perovskite solutions were spin-coated onto ITO/PTAA at 1000 rpm for 10 s, subsequently at 5000 rpm for 40 s. 250 μL CB was dripped onto the center of film at 12 s before the end of spin-coating. The deposited perovskite films were subsequently annealed on a hotplate at 110 ℃ for 20 min.

For the Fc-treated ($FcTc_2$, Fc_2Tc_2, and Fc_2Tc_2) devices, the Fc compound was prepared and dissolved in CB at an optimized concentration. The solution was stirred at room temperature until it became clear. Then, the solution was transferred to a N_2-filled glovebox before use. 100 μL of Fc compound solution was spin-coated on top of the as-prepared perovskite at 5000 rpm for 20 s, and annealed at 100 ℃ for 2 min. The spin-coating processes were all conducted at

room temperature in a N_2-filled glovebox with the contents of O_2 and H_2O < 10 ppm. Finally, 20 nm C60 at a rate of 0.5 Å s^{-1}, 6 nm BCP a rate of 0.5 Å s^{-1} and 100 nm silver electrode a rate of 1.0 Å s^{-1} were thermally evaporated, respectively, under high vacuum (< 4 × 10^{-6} Torr).

For device encapsulation, glass encapsulation covers with suitable dimensions and a thickness of approximately 0.7 mm were prepared. A high-hardness encapsulation adhesive (BONLE, model 9213) was applied evenly around the perimeter of the encapsulation glass cover. The adhesive-coated encapsulation cover was adhered onto the surface of the perovskite solar cell. A 30W UV lamp operating at 365 nm was used to irradiate the surface of the encapsulation cover for 30 seconds, allowing the encapsulation adhesive to cure. Finally, the edges of the encapsulation cover were sealed in contact with the device using encapsulation tape (made of polyisobutylene).

Characterization

The X-ray photo-electron spectroscopy (XPS) measurements were conducted by AXIS Supra XPS system. The surface and cross-section morphologies of the perovskite films were acquired by scanning electron microscopy (SEM) (QUATTROS, Thermal Fisher Scientific). All AFM-based characterizations (AFM, KPFM and EFM) were conducted through Bruker Dimension ICON, and Ti/Ir coated silicon tips (ASYELELC-01-R2) with a resonance frequency at ~ 58-97 KHz were used in Scanning Kelvin Probe Microscopy (SKPM) and Electrostatic Force Microscopy (EFM) imaging. The steady-state and time-resolved photoluminescence (PL) spectra were obtained by Edinburgh FLS980 applied with an excitation wavelength of 485 nm. The film thickness of perovskite was obtained by DektakXT stylus profiler. The UV-vis spectrometer (Perkin Elmer model Lambda 2S) system was used to record ultraviolet-visible (UV-vis) absorptions of films. Time-of-flight secondary-ion mass spectrometry (TOF-SIMS) measurements were performed using a TOF-SIMS V instrument (IONTOF GmbH, Cameca IMS 4F

NMR spectra were recorded at ambient temperature on a Bruker Avance 400 MHz spectrometer using 5 mm 507-pp NMR tubes and processed using MestReNova v14.3.2. NMR spectra were calibrated to the residual solvent peaks of $CDCl_3$ at 7.26 and 77.2 ppm for ^{1}H and $^{13}C\{^{1}H\}$, respectively. Peak assignment was aided through the use of COSY, edited HSQC and HMBC experiments. High resolution mass spectrometry data were recorded by direct injection on a Waters LCT time-of-flight (ToF) Mass Spectrometer using electrospray ionization (ESI) conducted by Malgorzata Puchnarewicz of the Mass Spectrometry Service at Imperial College London. Elemental analyses were analysed using a ThermoFlash 2000 Analyzer by the Elemental Analysis Service at London Metropolitan University. X-ray diffraction measurements were performed using Agilent Xcalibur 3 E ($FcTc_2$, Fc_2Tc_2 and Fc_2Tc_2-p2) and Xcalibur PX Ultra A (Fc_3Tc_2) diffractometers. The structures were solved and refined using the OLEX2,[3] SHELXTL[4] and SHELX-2013[5] program systems. The absolute structure of Fc_3Tc_2 was determined by use of the Flack parameter [$x^+ = +0.010(3)$]. CCDC 2280487 to 2280490.

Cyclic voltammetry analyses were conducted on a Gamry 600TM potentiostat to record the redox potentials associated with each ferrocene moiety. The samples were analysed in a cell comprising of glassy carbon working, platinum counter and silver pseudo reference electrode. The samples (2 mM) were dissolved in an electrolyte solution of tetrabutylammonium hexafluorophosphate (0.1 M) in anhydrous and degassed CH_2Cl_2, and analysed at scan rates of either 20, 50, 100, 250, 500 or 1000 mVs^{-1}. Each sample was referenced to a ferrocene/ferrocenium (Fc/Fc^+) internal reference and all scans corrected for solution resistance with values obtained from potentiostatic electrochemical impedance spectroscopy (EIS) measurements. UV-Vis analyses were conducted on a Agilent Technologies Cary 60 UV-Vis Spectrophotometer. All samples (5–1000 μM) were analysed at room temperature using a quartz cell with a path length of 1 cm in CH_2Cl_2.

The photovoltaic performance characteristics (*J-V* curves) of perovskite solar cells were conducted in a N_2-filled glovebox at room temperature using a Xenon lamp solar simulator (Enlitech, SS-F5, Taiwan). The light power was calibrated to 100 mW cm^{-2} by a silicon

reference cell (with a KG2 filter). Before *J-V* measurements, a 120-nm thick magnesium fluoride layer was deposited on the back of ITO substrate for transmittance enhancement. All the devices were measured using a Keithley 2400 source meter under a sweep mode of reverse scan (from 1.20 V to -0.01 V) and forward scan (from -0.01 V to 1.20 V) with the scan rate of 0.01 V s^{-1}, and the delay time was 10 ms. No pre-condition was needed before measurement. The active area was defined and characterized as 0.0419 cm^2 for the small-area and 1.0208 cm^2 for the centimetre-area by metal shadow mask. The stabilized power output was conducted by monitoring the stabilized current density output at the MPP bias (extracted from the reverse scan *J-V* curves). External quantum efficiency (EQE) measurements were carried out using a QE-R EQE system (Enlitech, Taiwan). Highly sensitive EQE was measured by an integrated system (PECT-600, Enlitech, Taiwan), where the photocurrent was amplified and modulated by a lock-in instrument. Electroluminescence (EL) quantum efficiency (EQE_{EL}) was conducted by applying an external voltage/current source through the instrument (ELCT-3010, Enlitech, Taiwan).

Stability tests.

The long-term operational stability was conducted by applying the perovskite solar cells under a 1 sun equivalent LED lamp in a N_2-filled glovebox (with the contents of O_2 and H_2O <10 ppm) at 65 °C. The PSCs were biased at maximum-power-point (MPP) voltage and the power output was tracked by using a multi-potentiostat (CHI1040C, CH Instruments, Inc.). A thermostatic control system was applied to keep the device at 65 °C. During the MPP test, the current density-voltage (*J-V)* curves of the devices were obtained every 12 h to get the proper loads for the MPP.

Density functional theory (DFT) calculations.

The *first principles* DFT simulations were performed with the Vienna Ab Initio Simulation Package (VASP 6.4) [6-8] to study the geometric and electronic structures of all the Fc compound treated $FAPbI_3$ perovskite series. Unless otherwise specified, the generalized gradient approximation exchange-correlation functional of Perdew-Burke-Ernzerhof (PBE)[8] was adopted in the DFT calculations. Due to the strong relativity effect of the heavy Pb atoms, we

adopted the spin-orbit coupling (SOC) effect in all the property calculations, except geometric optimization. The electronic constituents are 3d 3s 3p for Fe, 5d 6s 6p for Pb, 5p 6s for I, 2s 2p for O C and N, 3s 3p for S, and 1s for H. For all the quasi-2D Fc compound derived slabs, we adopted, 2 × 2 × 1 Γ-centered k-point grid, generated by the Monkhorst-Pack scheme, for detailed properties obtained with PBE functional. The projector augmented wave pseudopotentials with the cut-off energy of 600 eV were employed. Considering the interaction between the hydrogen atoms and high-electronegativity groups, the PBE with the DFT-D3 dispersion correction of Grimme with zero-damping [9-11] was applied to optimize the geometric structures. During the optimization of the geometries, all structures were allowed to relax to ensure that each atom was in mechanical equilibrium without any residual force larger than 10^{-4} eV/Å. For surface binding energy, we adopt the concept of $\varepsilon = \frac{E_{slab} - (\sum_i^n E_{i(comp)})}{2S}$, in which ε represents the surface binding energy value, E_{slab} is the optimized energy of the surface structure while $E_{i(comp)}$ means the optimized energy of every component of the hetero structure in independent vacuum boxes, the S term on the denominator represents the surface area of the structure.

NMR Spectra

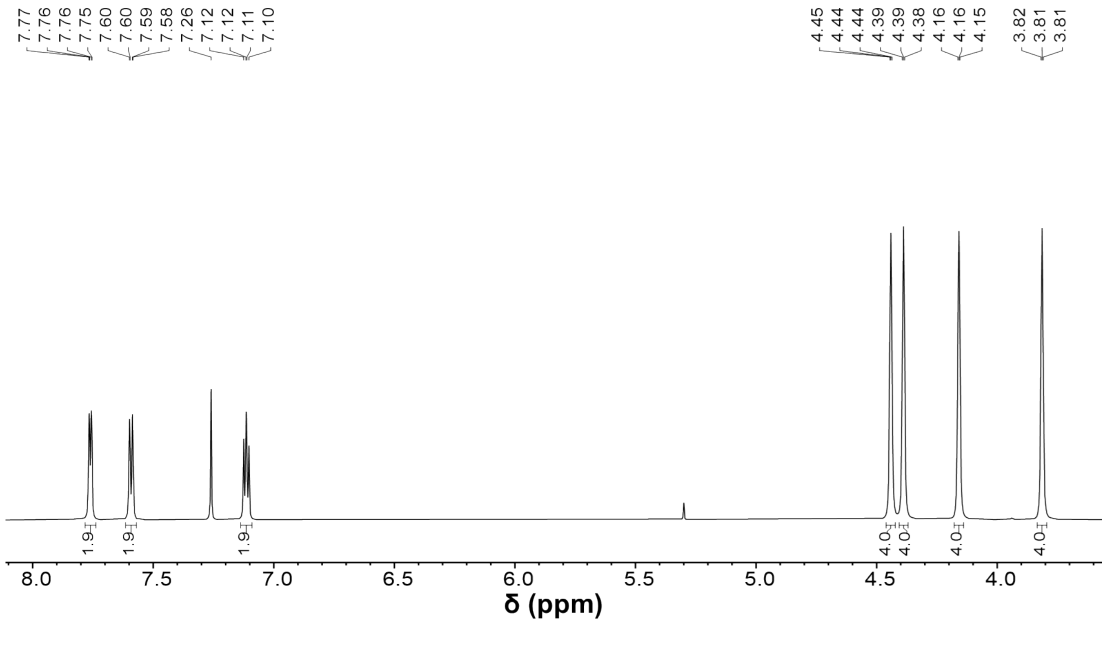

Fig. S1 ^1H NMR spectrum of Fc_2Tc_2 measured in $CDCl_3$.

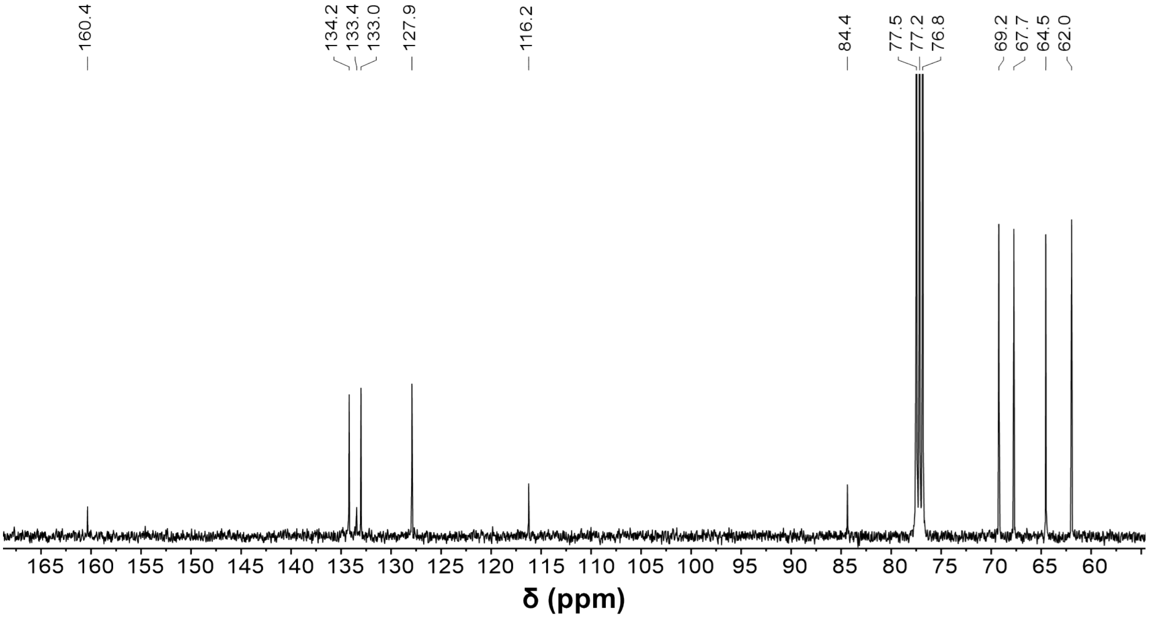

Fig. S2 $^{13}C\{^1H\}$ NMR spectrum of Fc_2Tc_2 measured in $CDCl_3$.

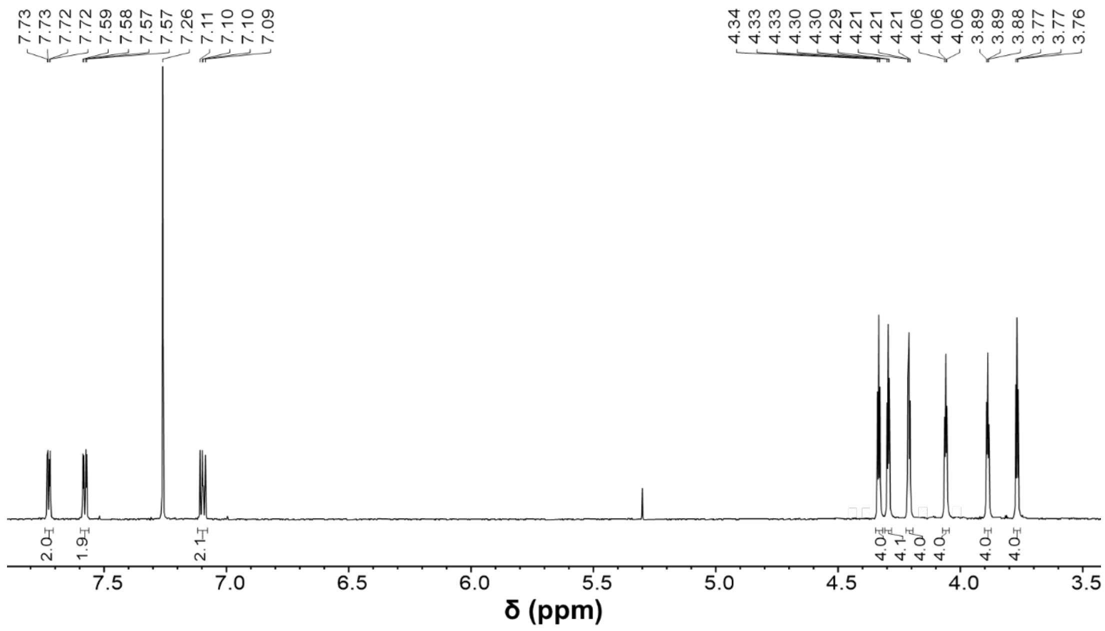

Fig. S3 ^1H NMR spectrum of Fc_3Tc_2 measured in $CDCl_3$.

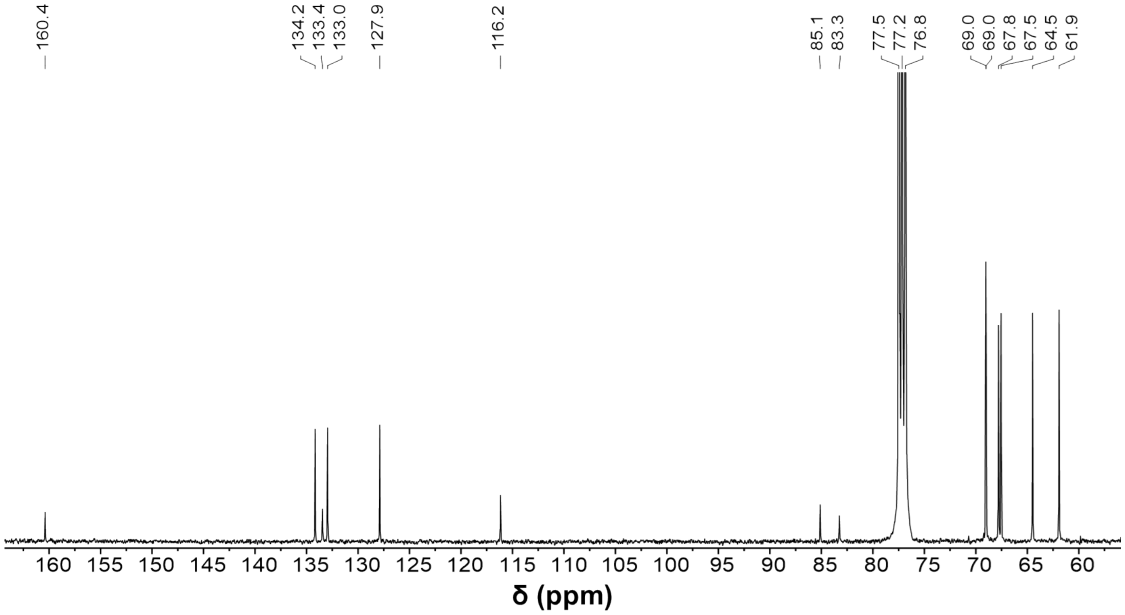

Fig. S4 $^{13}C\{^{1}H\}$ NMR spectrum of Fc_3Tc_2 measured in $CDCl_3$.

Note S1: Cyclic voltammetry data for Fc-derived compounds

Cyclic voltammetry experiments were performed for all three Fc-derived compounds which provide insight into their redox processes in solution. Fig. S5 shows a comparison cyclic voltammogram of the three ferrocenyl compounds at a scan rate of 100 mV s^{-1}. Each compound displays an oxidation and reduction event corresponding to the number of Fc moieties. By increasing the number of Fc units, the redox potential for the first, and subsequent events, decreases, suggesting that it is easier to perform a one-electron oxidation and reduction for $(Fc_3Tc_2)/(Fc_3Tc_2)^+$ than it is for $(Fc_2Tc_2)/(Fc_2Tc_2)^+$ and $(FcTc_2)/(FcTc_2)^+$. Plots of the peak current versus the square root of the scan rate (Figs. S7b, S9b, S9d, S11b, S11d, S11f) for each compound show linear fits, indicating reversible electron transfer for each redox event. Table S1 summarizes the findings for each Fc-derived compound.

The cyclic voltammogram of $FcTc_2$ shows a single redox event corresponding to the $(FcTc_2)/(FcTc_2)^+$ redox couple (Fig. S6), whereas the cyclic voltammogram for Fc_2Tc_2 shows two reversible redox events corresponding to the $(Fc_2Tc_2)/(Fc_2Tc_2)^+$ and $(Fc_2Tc_2)^+/(Fc_2Tc_2)^{2+}$ redox couple (Fig. S8). Although the cyclic voltammogram of Fc_3Tc_2 shows three redox events (Fig. S10), the third, corresponding to the $(Fc_3Tc_2)^{2+}/(Fc_3Tc_2)^{3+}$ redox couple, is less clear and features abnormal oxidation and reduction peaks. When isolating the first two redox events (Fig. S12), both oxidation and reduction peaks for the $(Fc_3Tc_2)^{2+}/(Fc_3Tc_2)^{3+}$ redox couple disappear, showing a voltammogram similar to that of Fc_2Tc_2. This indicates that the oxidation and reduction events of the $(Fc_3Tc_2)^{2+}/(Fc_3Tc_2)^{3+}$ couple are related to each other, albeit with a large distance between potential peaks (ΔE = 330 mV). This event is presumably due to the central Fc moiety, and its difficulty to reduce once oxidised may be caused by Fc_3Tc_2 becoming trapped in the +3 oxidation state. The difficulty to reduce this molecule from +3 to +2 may also contribute to its lower efficiency when applied in inverted perovskite cells as this may prevent efficient transfer of electrons.

By performing square wave voltammetry (SWV) experiments (Fig. S13 a–c), each redox event can be displayed which can be correlated to the oxidation state of each molecule. Although the SWV for Fc_3Tc_2 (Fig. S13 c) shows three distinct peaks, indicating three redox events, the last

peak is distinctly different to the other two associated with the central Fc unit and the challenging reversibility of the $(Fc_3Tc_2)^{2+}/(Fc_3Tc_2)^{3+}$ redox couple.

Cyclic Voltammetry

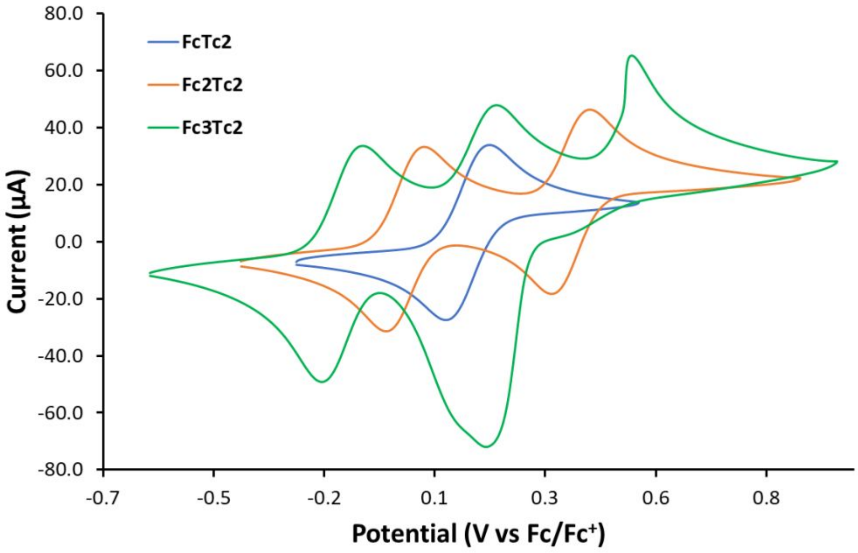

Fig. S5 Comparison cyclic voltammogram of $FcTc_2$, Fc_2Tc_2 and Fc_3Tc_2 measured at 100 mV s^{-1}.

Table S1 Cyclic voltammogram data (reported in mV and referenced to the Fc/Fc^{+} redox couple) for $FcTc_2$, Fc_2Tc_2 and Fc_3Tc_2 measured at 100 mV s^{-1}.

	E_{pa}	E_{pc}	$E_{1/2}$	ΔE	i_{pa}/i_{pc}
**$FcTc_2$**	174	76	125	98	0.99
**Fc_2Tc_2**	26	–60	–17	86	0.68
	400	314	357	86	2.34
**Fc_3Tc_2**	–113	–205	–159	93	0.76
	190	116	153	74	0.62
	496	165	331	330	0.72

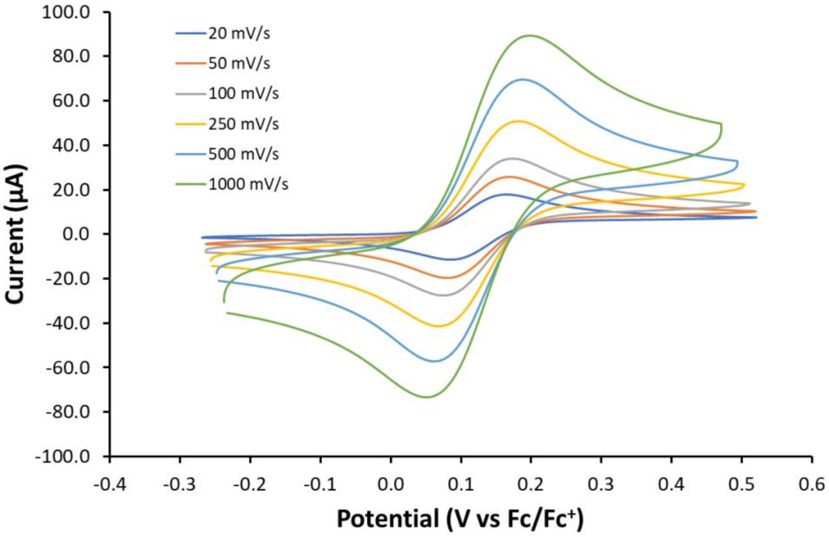

Fig. S6 Cyclic voltammogram of $FcTc_2$ with varying scan rates.

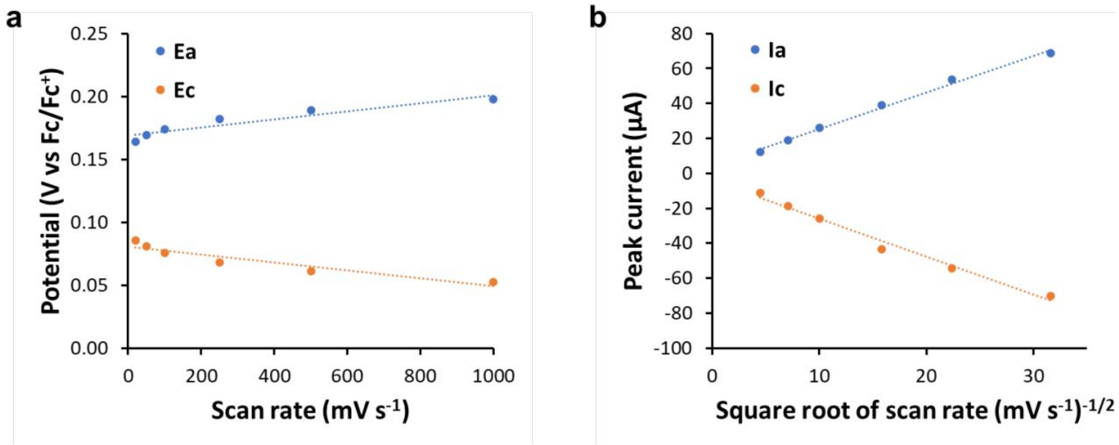

Fig. S7 a and b, Plot of potential versus scan rate (**a**) and peak current versus the square root of the scan rate (**b**) with linear fits for $FcTc_2$.

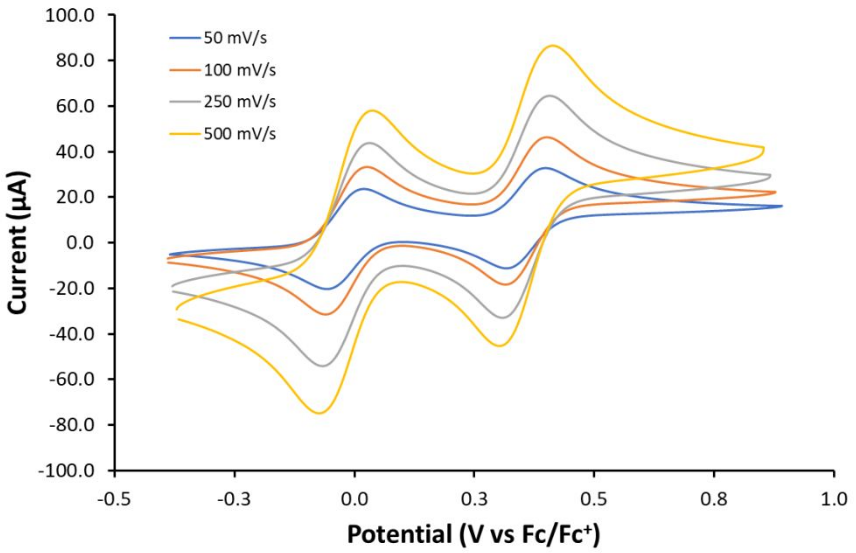

Fig. S8 Cyclic voltammogram of Fc_2Tc_2 with varying scan rates.

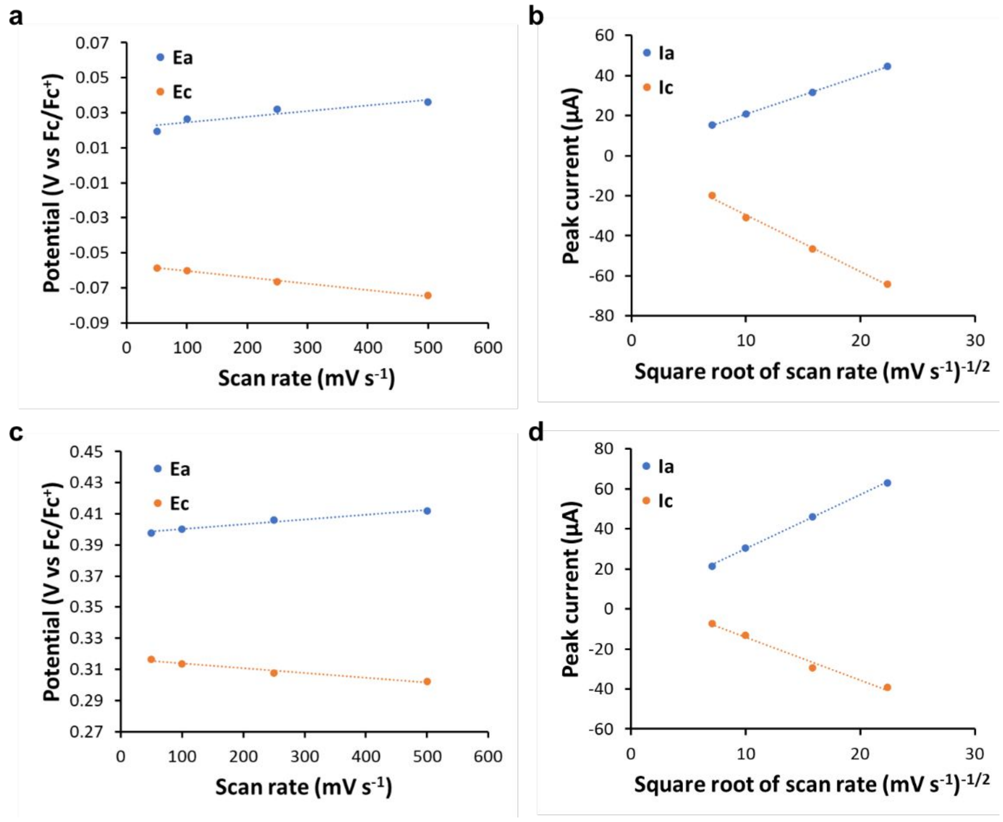

Fig. S9 a and b, Plot of potential versus scan rate (**a**) and peak current versus the square root of the scan rate (**b**) with linear fits for the **first redox** event for Fc_2Tc_2. **c and d**, Plot of potential versus scan rate (**c**) and peak current versus the square root of the scan rate (**d**) with linear fits for the **second redox** event for Fc_2Tc_2.

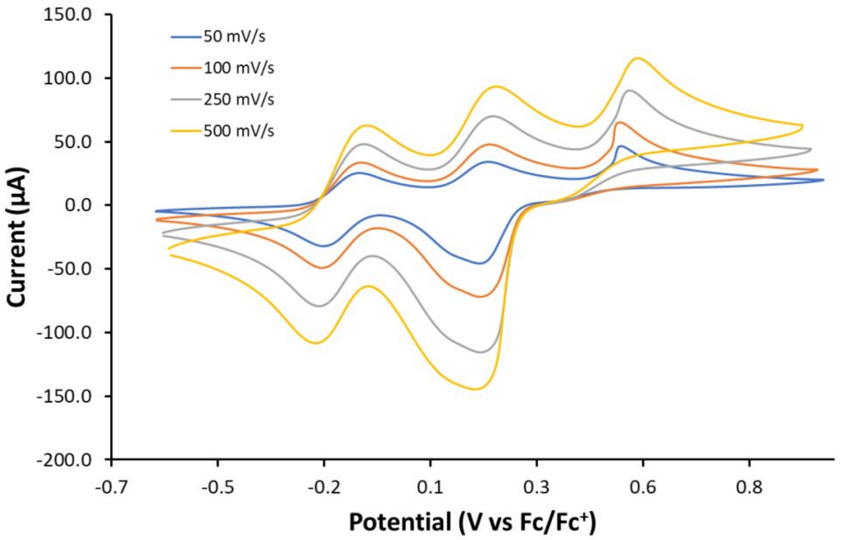

Fig. S10 Cyclic voltammogram of Fc_3Tc_2 with varying scan rates.

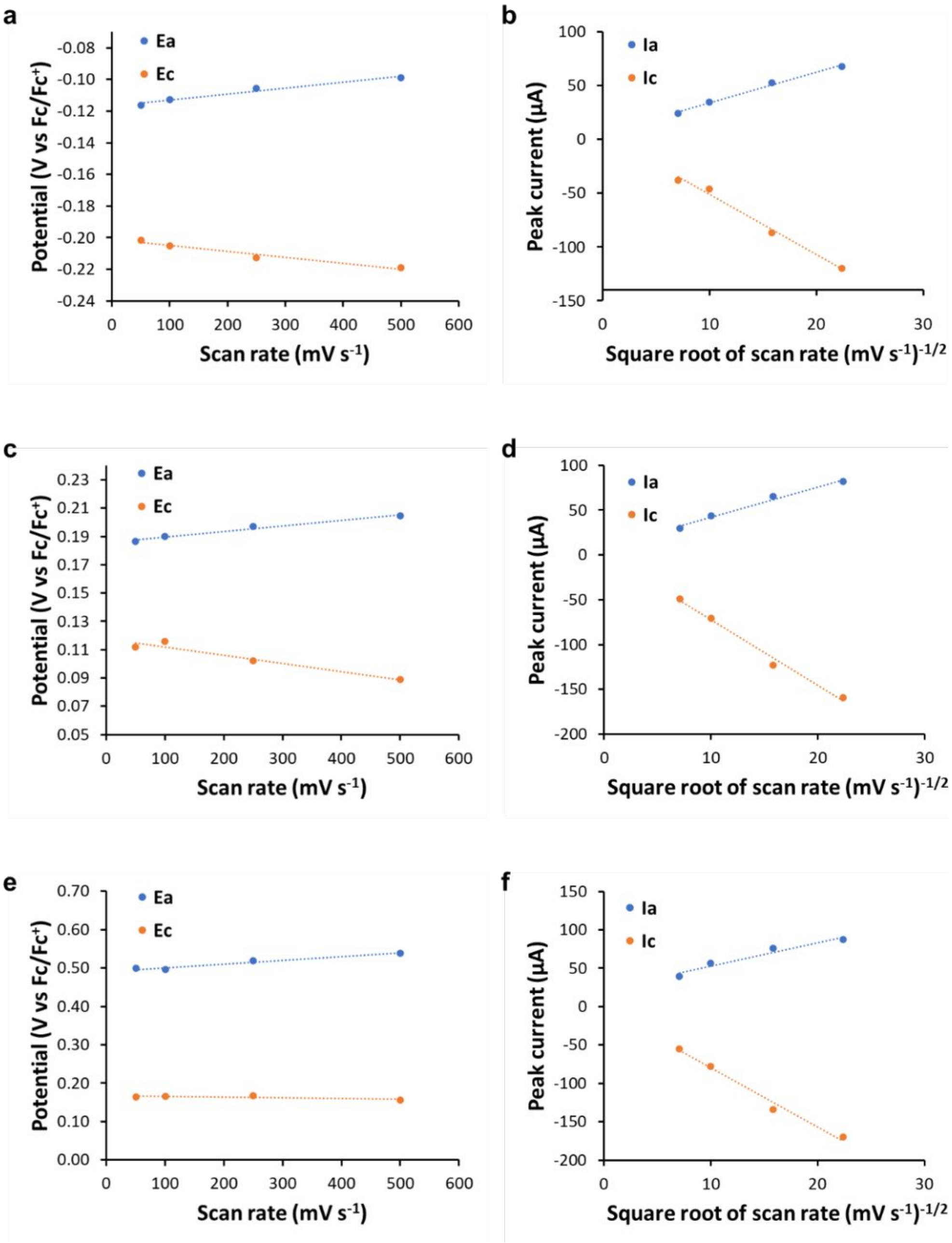

Fig. S11 a and b, plot of potential versus scan rate (**a**) and peak current versus the square root of the scan rate (**b**) with linear fits for the **first redox** event for Fc_3Tc_2; **c and d,** plot of potential versus scan rate (**c**) and peak current versus the square root of the scan rate (**d**) with linear fits for the **second redox** event for Fc_3Tc_2; **e and f,** plot of potential versus scan rate (**e**) and peak current versus the square root of the scan rate (**f**) with linear fits for the **third redox** event for Fc_3Tc_2.

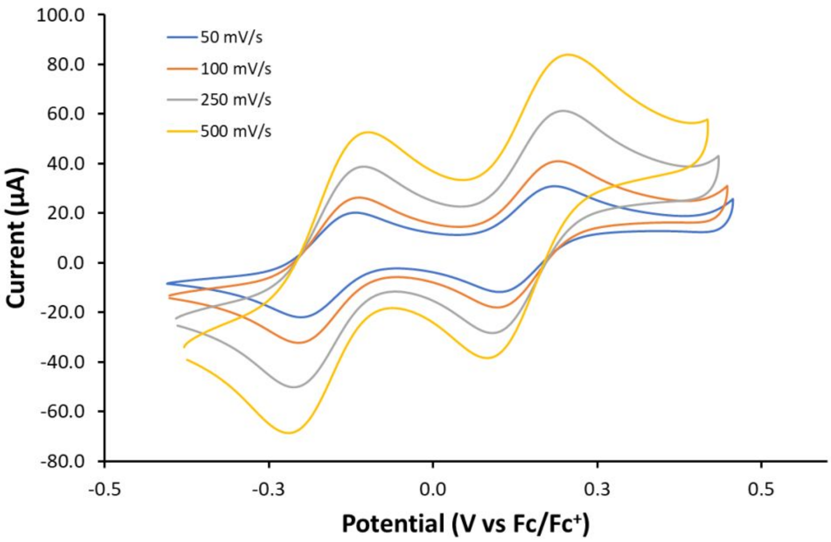

Fig. S12 Cyclic voltammogram of the first two redox events of Fc_3Tc_2 with varying scan rates.

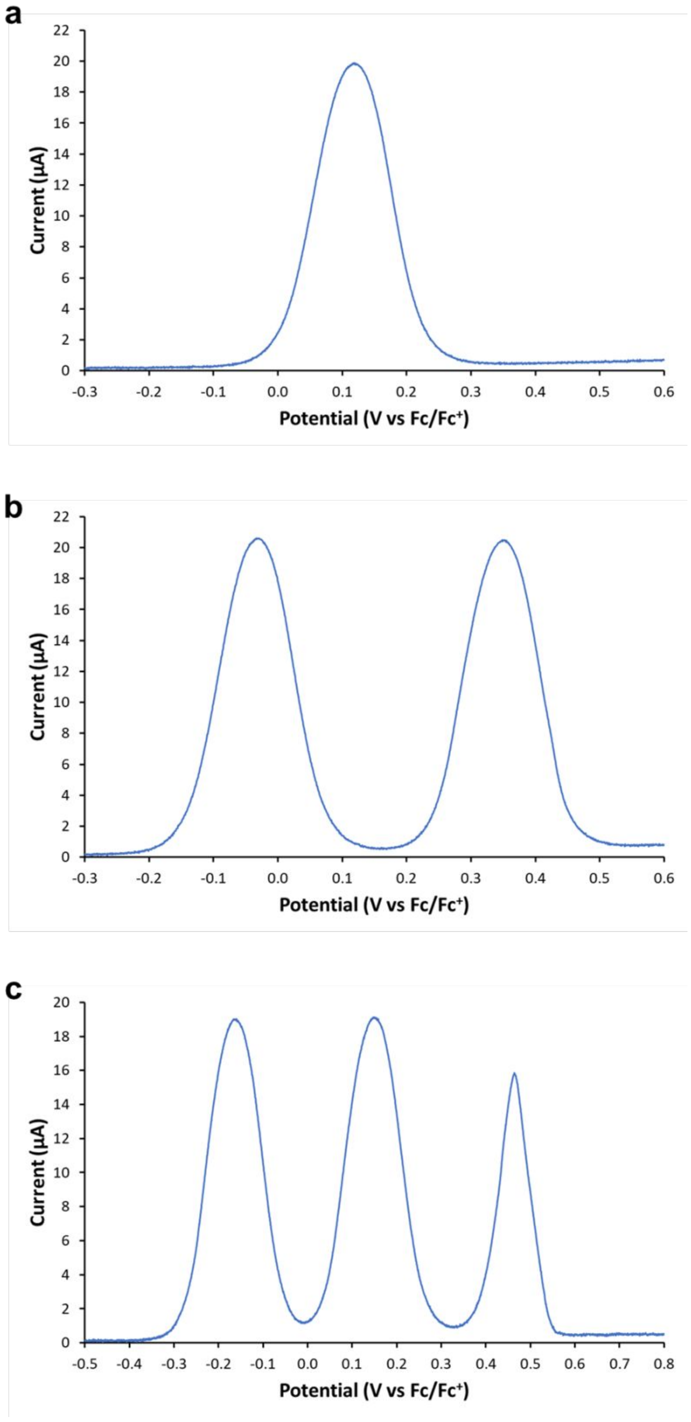

Fig. S13 a–c, Square wave voltammograms of $FcTc_2$ (**a**), Fc_2Tc_2 (**b**) and Fc_3Tc_2 (**c**).

UV-Vis

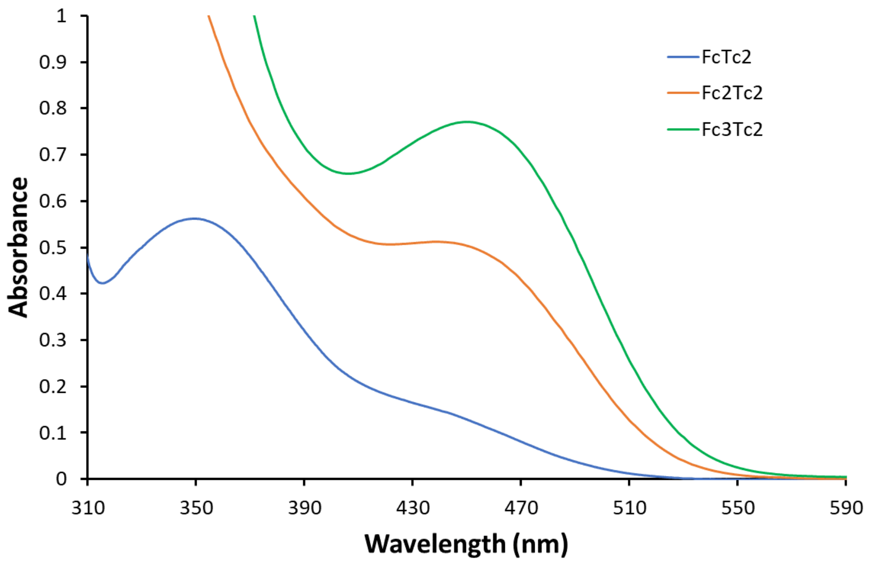

Fig. S14 Comparison UV-Vis absorption spectra of $FcTc_2$, Fc_2Tc_2 and Fc_3Tc_2 measured in CH_2Cl_2 (500 μM).

X-ray Diffraction

Table S2 Crystal Data, Data Collection and Refinement Parameters for the structures of $FcTc_2$, Fc_2Tc_2, Fc_2Tc_2-p2 and Fc_3Tc_2.

	$FcTc_2$	Fc_2Tc_2	Fc_2Tc_2-p2	Fc_3Tc_2
**Formula**	$C_{20}H_{14}FeO_4S_2$	$C_{30}H_{22}Fe_2O_4S_2$	$C_{30}H_{22}Fe_2O_4S_2$	$C_{40}H_{30}Fe_3O_4S_2$
**Solvent**	—	—	—	—
**Formula weight**	438.28	622.29	622.29	806.31
**Color, habit**	orange blocky	orange platy	orange blocks	red plates
**Temperature / K**	173	173	173	173
**Crystal system**	triclinic	monoclinic	monoclinic	monoclinic
**Space group**	*P*–1 (no. 2)	$P2_1/c$ (no. 14)	$P2_1/c$ (no. 14)	$P2_1$ (no. 4)
a **/ Å**	10.3965(9)	5.9894(3)	12.1753(3)	5.96639(7)
b **/ Å**	11.0782(8)	15.0252(5)	14.7071(4)	16.10950(19)
c **/ Å**	16.7581(13)	13.9197(6)	14.1536(4)	16.45896(16)
**α / deg**	80.375(6)	90	90	90
**β / deg**	86.696(7)	94.094(3)	91.748(3)	93.6293(9)
**γ / deg**	68.702(8)	90	90	90
V **/ Å^3**	1773.0(3)	1249.46(9)	2533.23(12)	1578.79(3)
Z	4[c]	2[d]	4	2
D_c **/ g cm^{-3}**	1.642	1.654	1.632	1.696
**Radiation used**	Mo-Kα	Mo-Kα	Mo-Kα	Cu-Kα
**μ / mm^{-1}**	1.111	1.366	1.347	12.512
**No. of unique reflns**				
**measured (R_{int})**	8073 (0.0355)	2624 (0.0271)	5585 (0.0329)	5936 (0.0448)
**obs, $\|F_o\| > 4\sigma(\|F_o\|)$**	5832	2240	4436	5621
**Completeness (%)[a]**	97.5	99.7	99.9	100
**No. of variables**	572	193	385	509
**R_1(obs), wR_2(all)[b]**	0.0417, 0.0698	0.0317, 0.0762	0.0423, 0.1034	0.0280, 0.0684
**CCDC code**	2280487	2280488	2280489	2280490

[a] Completeness to 0.84 Å resolution. [b] $R_1 = \Sigma||F_o| - |F_c||/\Sigma|F_o|$; $wR_2 = \{\Sigma[w(F_o^2 - F_c^2)^2] / \Sigma[w(F_o^2)^2]\}^{1/2}$; $w^{-1} = \sigma^2(F_o^2) + (aP)^2 + bP$. [c] There are two crystallographically independent molecules. [d] The complex has crystallographic C_i symmetry.

Note S2: The X-ray crystal structure of Fc compounds

The X-ray crystal structure of $FcTc_2$

The crystal of $FcTc_2$ that was studied was found to be a two-component twin in a *ca*. 65:35 ratio, with the two lattices related by the approximate twin law [–1.00 0.00 0.00 0.00 –1.00 0.00 0.02 –0.52 1.00]. The structure was found to contain two crystallographically independent complexes ($FcTc_2$-A and $FcTc_2$-B) in the asymmetric unit. The C13-and C20-based thiophene groups in both independent complexes were found to be disordered, and in each case two orientations were identified of *ca*. 79:21, 67:33, 61:39 and 59:41% occupancy respectively. The geometries of each pair of orientations were optimised, the thermal parameters of adjacent atoms were restrained to be similar, and only the non-hydrogen atoms of the major occupancy orientations were refined anisotropically (those of the minor occupancy orientations were refined isotropically).

The X-ray crystal structure of Fc_2Tc_2

The structure of Fc_2Tc_2 was found to sit across a centre of symmetry at the middle of the C6–C6A bond between the two central Cp rings. The unique C13-based thiophene group was found to be disordered. Two orientations were identified of *ca*. 74 and 26% occupancy, their geometries were optimised, the thermal parameters of adjacent atoms were restrained to be similar, and only the non-hydrogen atoms of the major occupancy orientation were refined anisotropically (those of the minor occupancy orientation were refined isotropically).

The X-ray crystal structure of Fc_2Tc_2-p2

The C23-and C30-based thiophene groups in the structure of Fc_2Tc_2-p2 were both found to be disordered, and in each case two orientations were identified of *ca*. 78:22 and 68:32% occupancy respectively. The geometries of each pair of orientations were optimised, the thermal parameters of adjacent atoms were restrained to be similar, and only the non-hydrogen atoms of the major occupancy orientations were refined anisotropically (those of the minor occupancy orientations were refined isotropically).

The X-ray crystal structure of Fc_3Tc_2

The O31-based thiophene ester group in the structure of Fc_3Tc_2 was found to be disordered. Three orientations were identified of ca. 48, 26 and 26% occupancy, their geometries were optimised, the thermal parameters of adjacent atoms were restrained to be similar, and only the non-hydrogen atoms of the major occupancy orientation were refined anisotropically (those of the minor occupancy orientations were refined isotropically). The absolute structure of Fc_3Tc_2 was determined by use of the Flack parameter [$x^+ = +0.010(3)$].

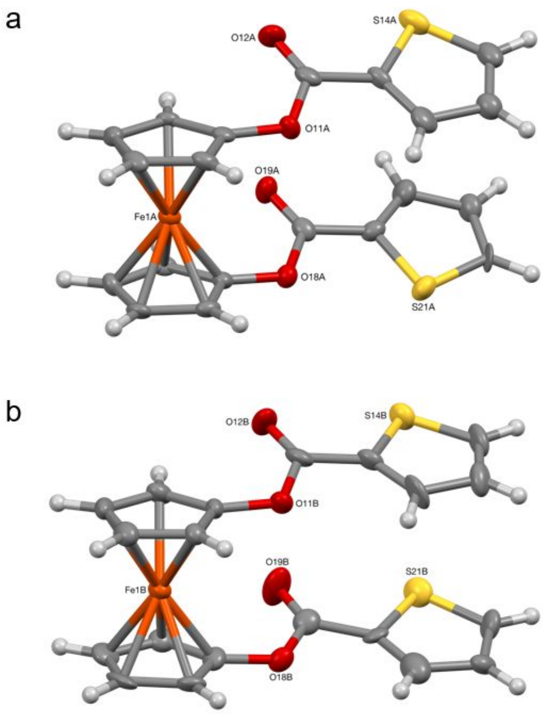

Fig. S15 a, The structure of $FcTc_2$-A, one of the two independent complexes present in the crystal of $FcTc_2$ (50% probability ellipsoids). **b**, The structure of $FcTc_2$-B, one of the two independent complexes present in the crystal of $FcTc_2$ (50% probability ellipsoids).

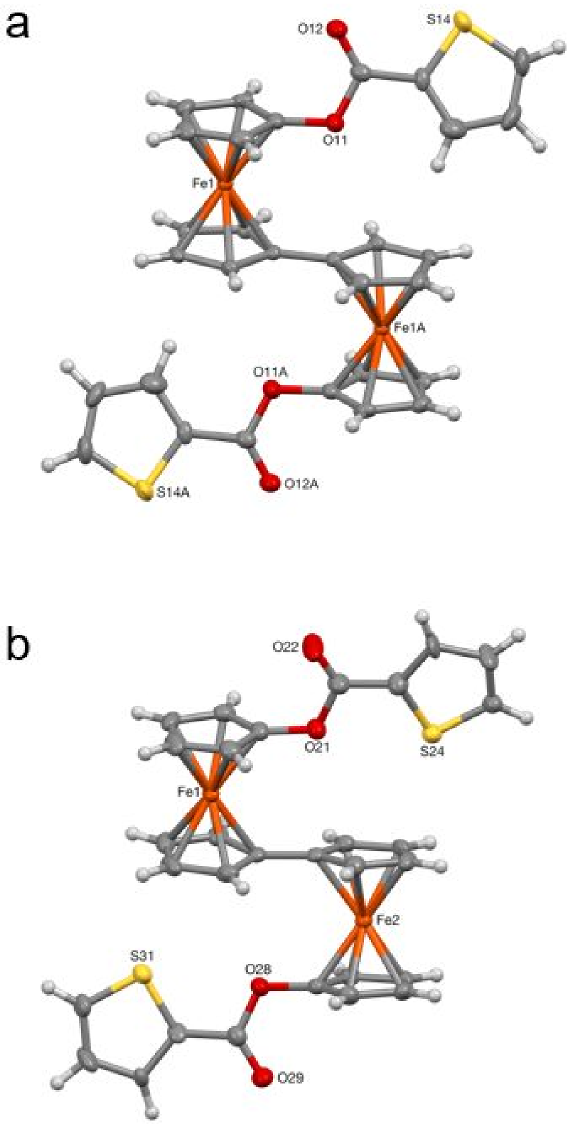

Fig. S16 a. The structure of the C_i-symmetric complex present in the crystal of Fc_2Tc_2 (50% probability ellipsoids). **b**. The crystal structure of Fc_2Tc_2-p2 (50% probability ellipsoids).

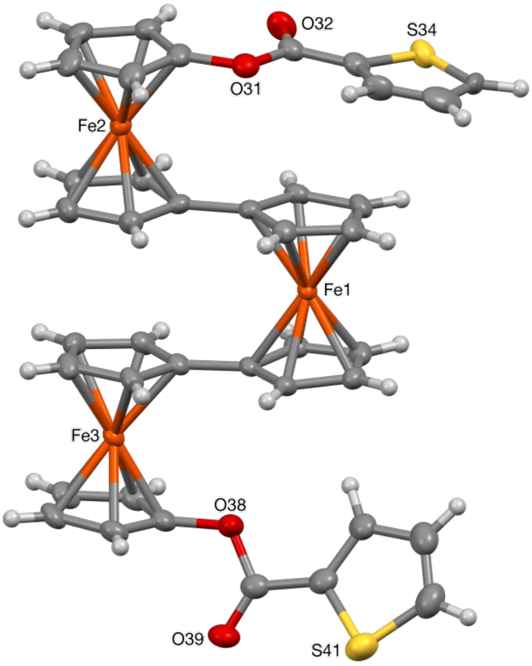

Fig. S17 The crystal structure of Fc_3Tc_2 (50% probability ellipsoids).

Note S3: Structural evolution of perovskites under the influence of Fc compounds

In Fig. S18, *$FcTc_2$* is attached to the perovskite surface through a Pb-O bond with a bond length of 3.24 Å. $FcTc_2$ causes distortion of the localized Pb-I octahedron, resulting in one side of the Pb-I bond length being elongated to 3.64 Å, while another side being shortened to 3.05 Å. The $FcTc_2$-attached surface bond angle I-Pb-I becomes 169.6° accordingly.

For the larger *Fc_2Tc_2*, it is connected to the perovskite surface through a Pb-O bond with a bond length of 2.56 Å, forming a coordination bond. Fc_2Tc_2 causes stronger distortion of the Pb-I octahedron, resulting in one side of the Pb-I bond length being elongated to 3.54 Å, while another side being shortened to 3.11 Å. The surface bond angle I-Pb-I becomes 152.6° accordingly. Moreover, the distorted Pb-I octahedron leads to the Pb atoms being uplifted towards the Fc_2Tc_2 and to be located higher than the crystal surface.

For *Fc_3Tc_2*, the bond length of the Pb-O bond is 2.98 Å. The Pb-I bond length is elongated to 3.68 Å on one side, while another side is shortened to 3.16 Å. The surface bond angle I-Pb-I becomes 155.2° accordingly.

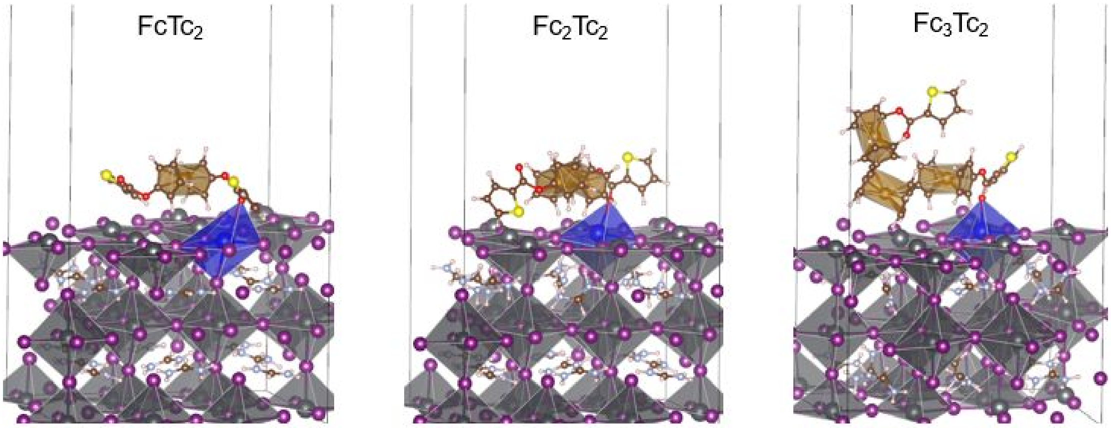

Fig. S18 Structural simulation of the mono- and poly-ferrocenyl compounds-treated perovskite surface.

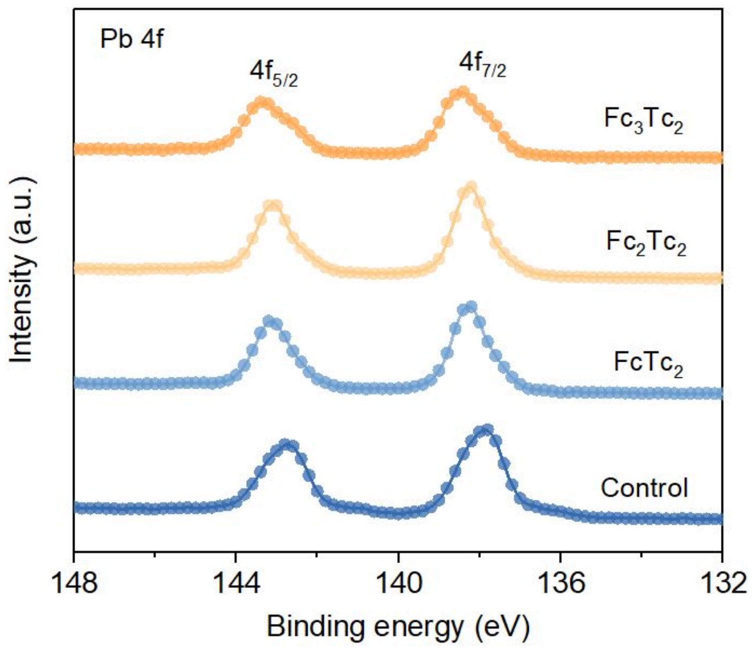

Fig. S19 XPS spectra of elemental Pb in perovskite films with the different Fc compounds.

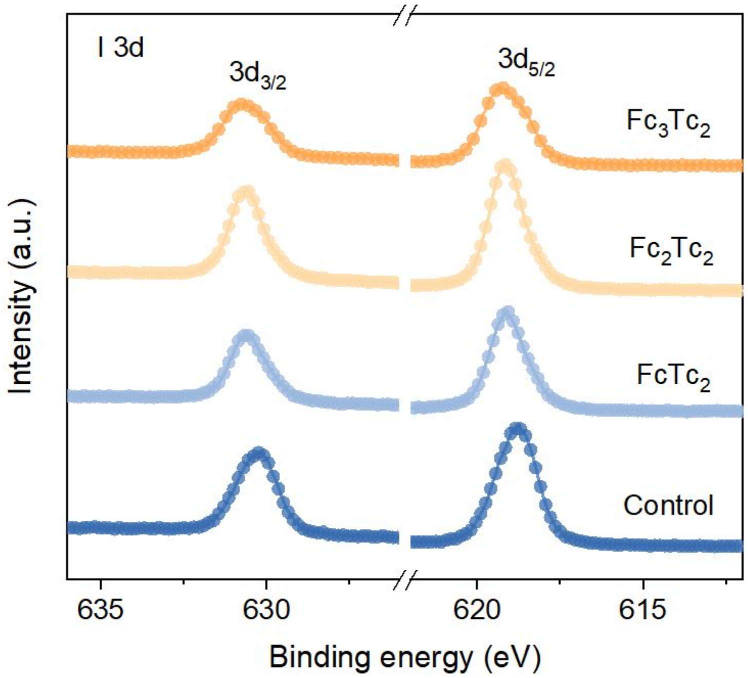

Fig. S20 XPS spectra of elemental I in perovskite films with the different Fc compounds.

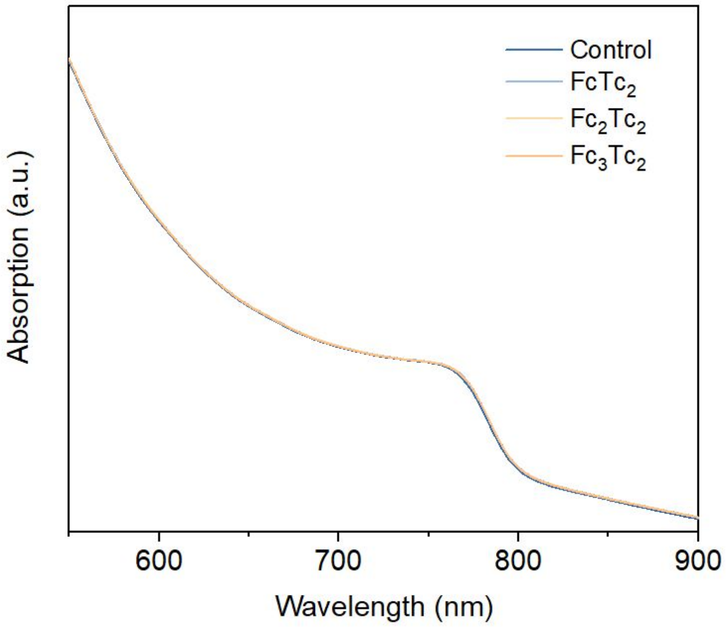

Fig. S21 UV-vis absorption spectra of perovskite films with the different Fc compounds**.**

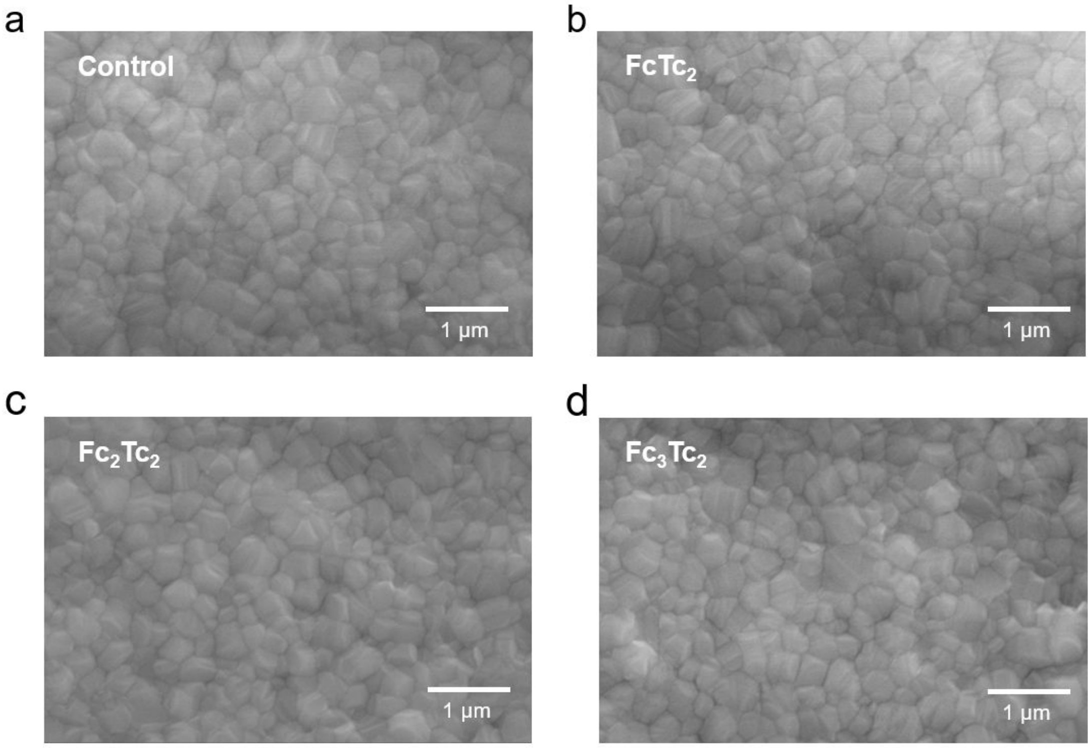

Fig. S22 SEM images of perovskite films with the different Fc compounds**.**

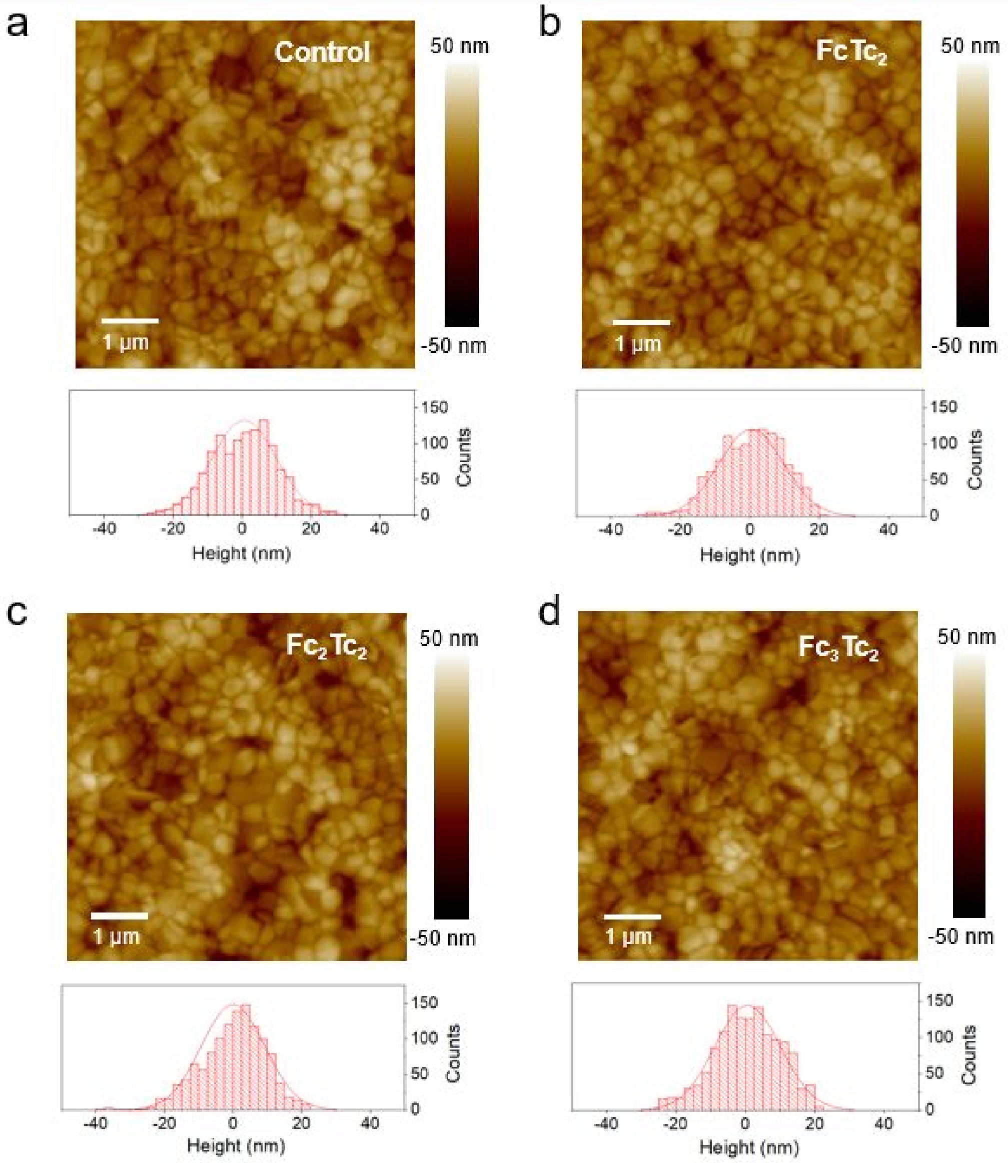

Fig. S23 AFM images of perovskite films with the different Fc compounds.

Note S4: Electronic structure of perovskites under the influence of poly-ferrocenes.

In Fig. S24, the charge density distributions of I and Pb are depicted, where the changes in I-5p contributions affect the valence band maximum (VBM), while the changes in Pb-6p contributions affect the conduction band minimum (CBM). The Fc_2Tc_2 modification leads to the changes in the charge distribution of Pb and I on the perovskite surface. Specifically, after modification with Fc_2Tc_2, the Pb-I octahedral coordinated to Tc exhibits an increased charge density surrounding the I, and a decrease in the charge density surrounding the Pb. The charge redistribution results in a shift of the energy levels of the VBM and CBM, which ultimately affects the electronic properties of the perovskite in Fig. S25.

Figs. S26 and S27 present the projection density of state (pDOS) of Pb and I in three different states: bulk, surface, and coordinated via bi-ferrocenyl Fc_2Tc_2. The pDOS reveals how the electronic states of the atoms in the material contribute to the overall electronic structure. For Pb, the uncoordinated Pb on the surface of the perovskite material lowers the position of the CBM, which stems from the peak at the CBM formed by the vacant orbital of the undercoordinated Pb in $[PbI_5]^{3-}$. However, when coordinated by Fc_2Tc_2, the CBM raises by about 0.2 eV, which restores it to the position of bulk Pb. This change indicates that the electronic configuration returns to a state closer to bulk $[PbI_6]^{4-}$, demonstrating the effective passivation of undercoordinated Pb. For pDOS of I, the coordination of Fc_2Tc_2 increases the VBM by about 0.2 eV and forms a strong peak at the VBM. This change originates from the extra two electrons squeezed into the VBM after forming a resonance hybrid, revealing the presence of activated electrons on the perovskite surface due to the coordination effect of the poly-ferrocenyl species.

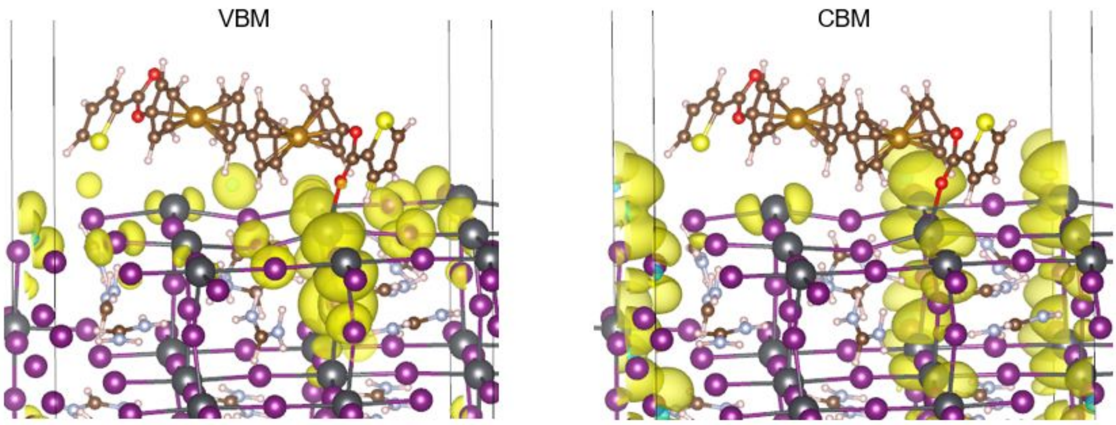

Fig. S24 Charge density distribution of perovskite surface modified with Fc_2Tc_2

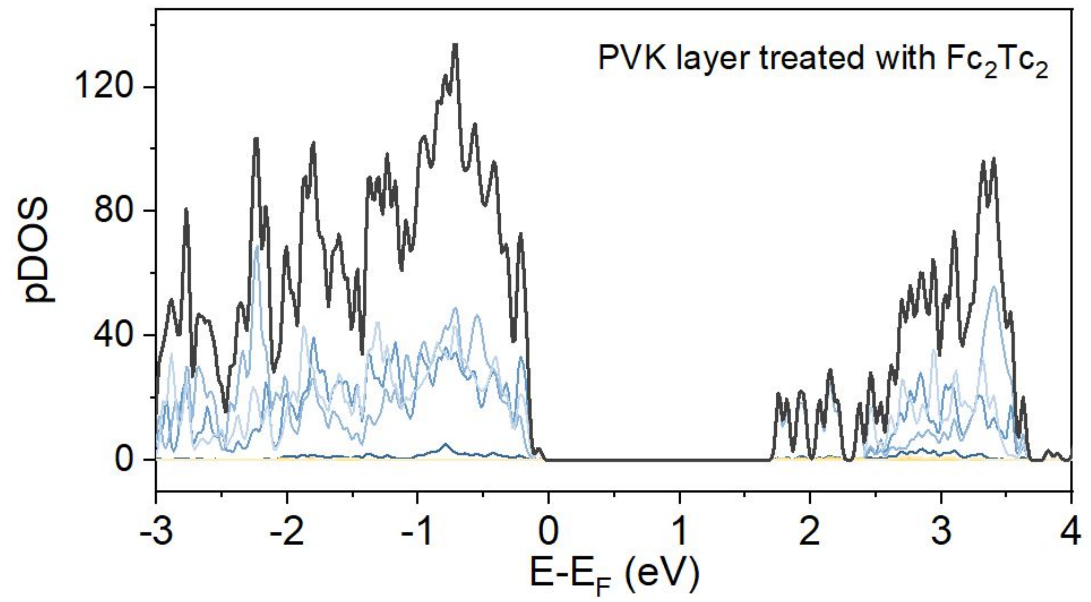

Fig. S25 pDOS plots of the perovskite layer after modifying with Fc_2Tc_2

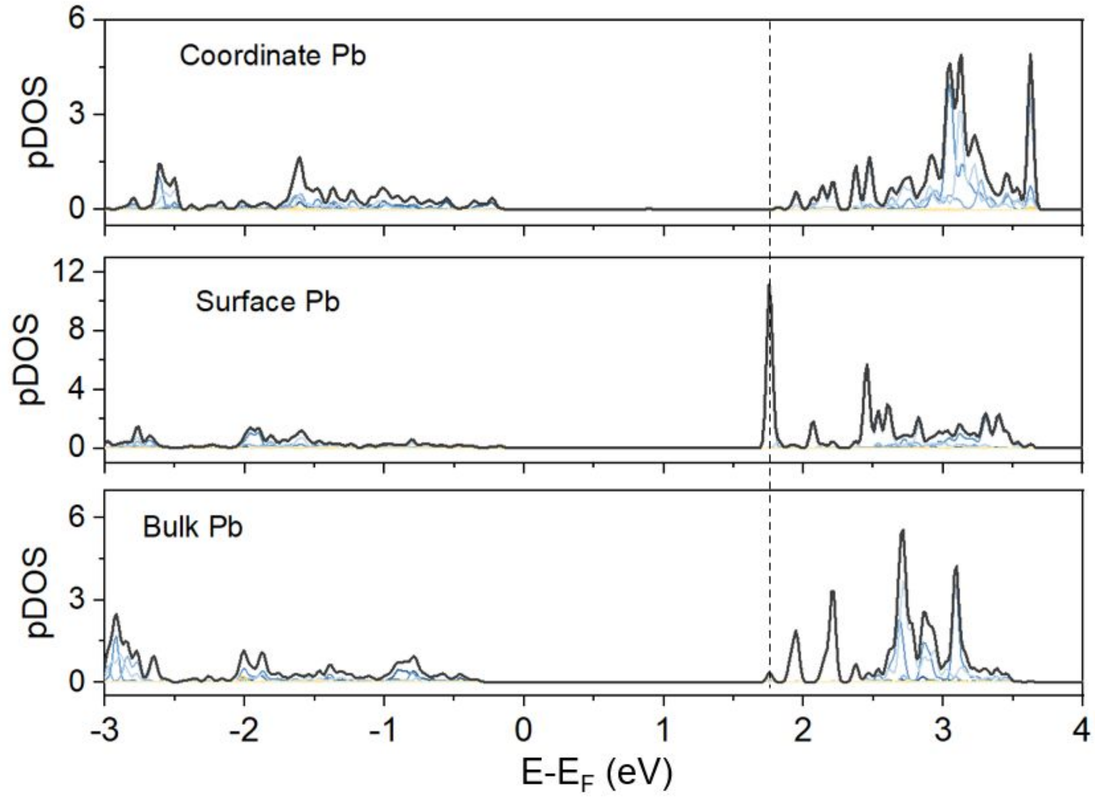

Fig. S26 pDOS plots of the bulk Pb, surface Pb and coordinated Pb via Fc_2Tc_2.

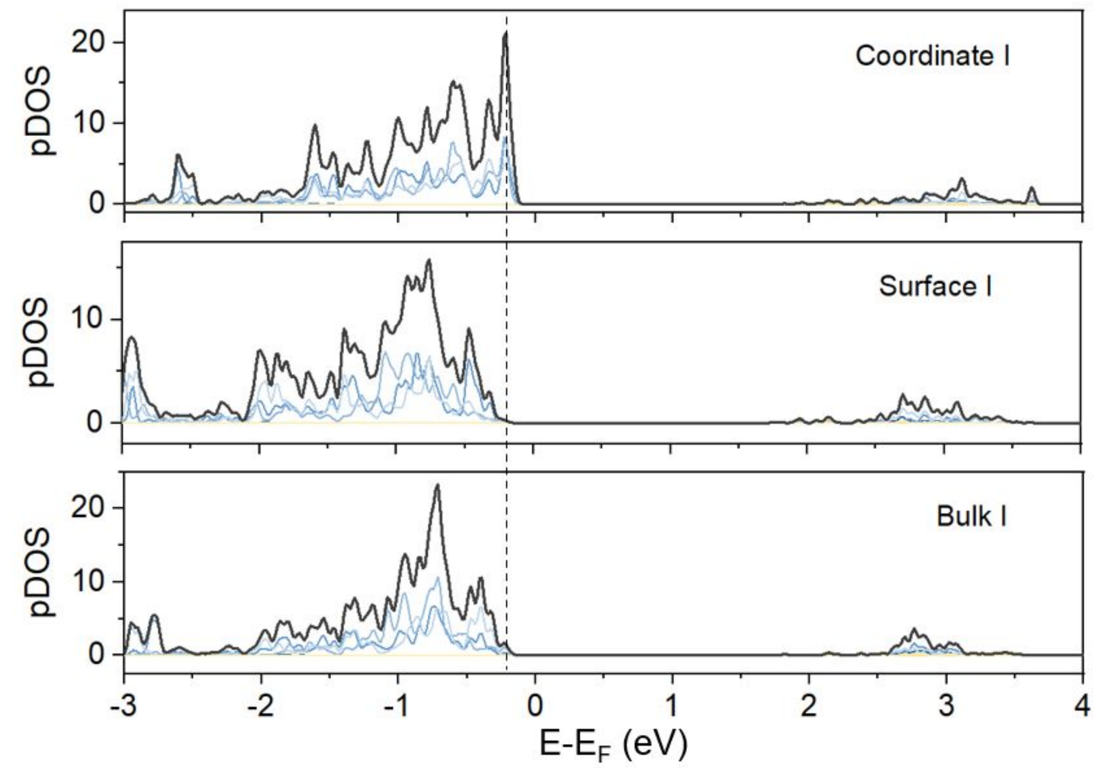

Fig. S27 pDOS plots of the bulk I, surface I and coordinated I via Fc_2Tc_2.

Note S5: Electrostatic force microscopy (EFM)

EFM provides a powerful tool to discover direct charge transfer of mixed electron systems. In the test protocol of EFM, a bias voltage (-3 to 3 V with a 1.5 V step) is applied on the tip of the probe to allow extraction of Coulomb forces. Figures 2A and 2B show the phase shift mapping of the entire scan area at different bias voltages integrated in one image for comparison. We show the statistics of phase angle under different bias voltages in Figs. S28 and S29 by counting the data on Fig. 2A and 2B. Further, we show the statistical mean in Figs. 2C and 2D, and perform a parabolic fit to it. The negative shift of the fitted parabolic axis of symmetry represents the negative charge induced at a surface point or region.

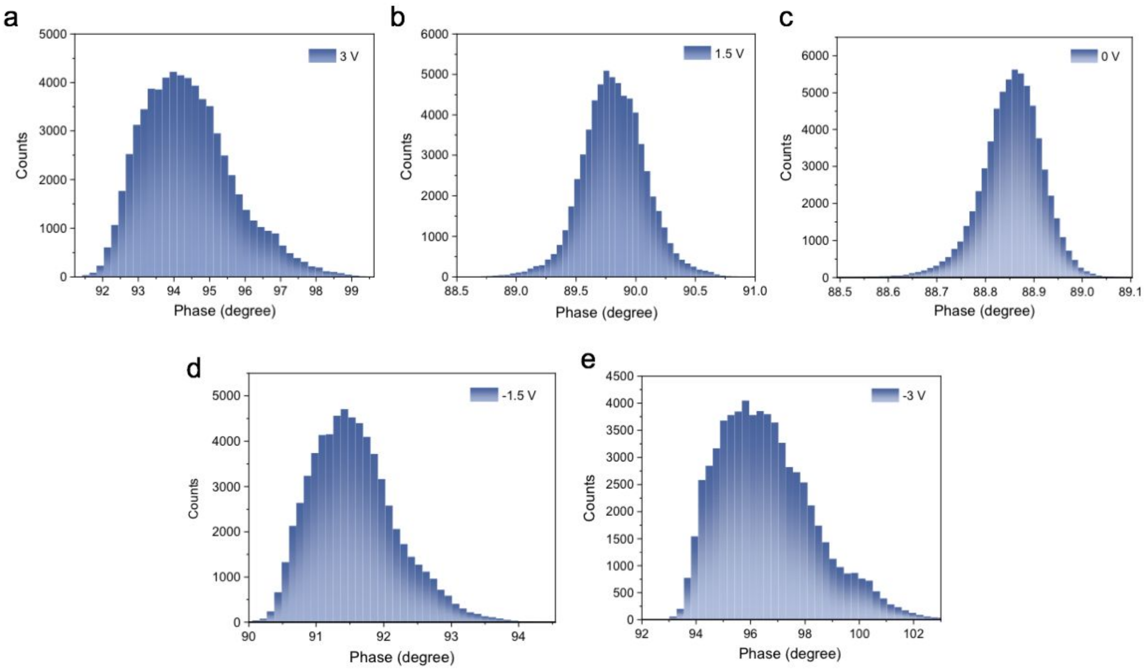

Fig. S28 Statistics of phase angle of pristine perovskite films under different applied bias voltages.

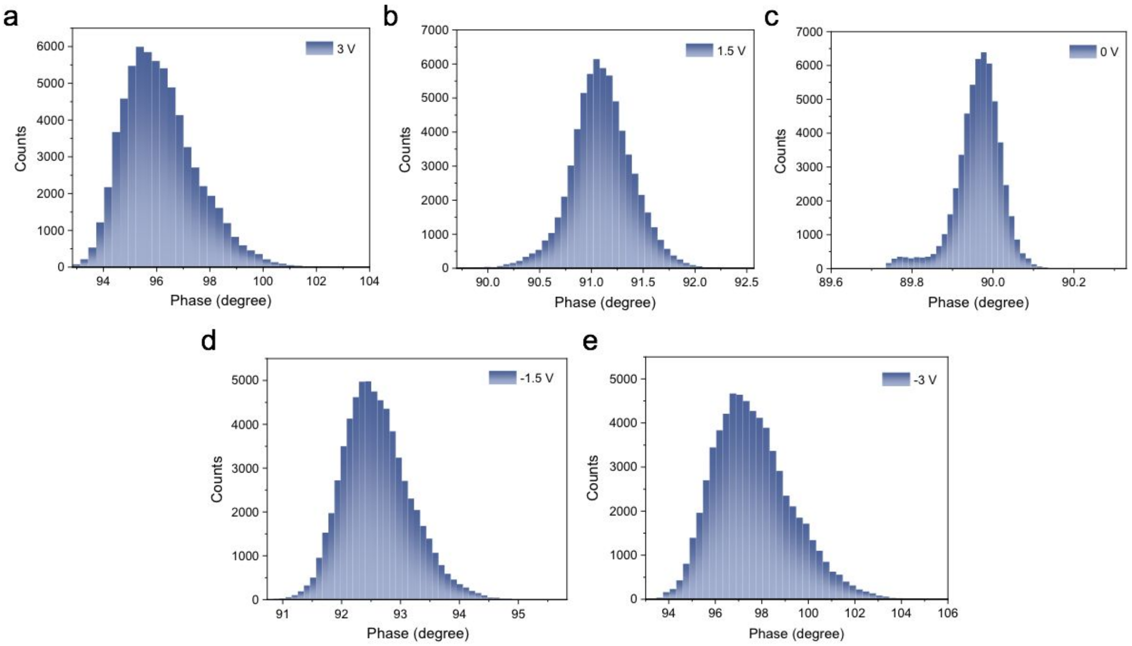

Fig. S29 Statistics of phase angle of Fc_2Tc_2-modified perovskite films under different applied bias voltages.

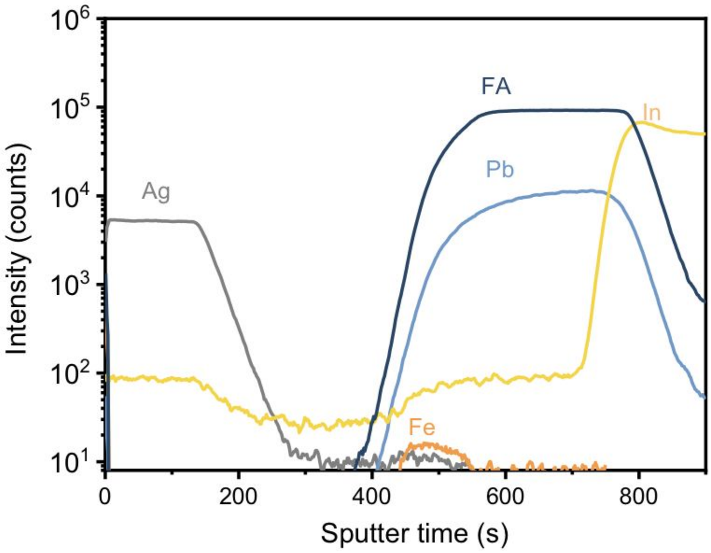

Fig. S30 TOF-SIMS plots of Fc_2Tc_2-modified perovskite solar cells.

Note S6: Space charge limited current (SCLC) measurements.

The electron-only devices with the FTO/TiO_2/perovskite/Fc/C60/BCP/Ag structure were prepared to calculate the defect density (*N*). In the SCLC regime, the current is dominated by charge carriers injected from the contacts and the current-voltage characteristics become quadratic ($I \sim V^2$). Fig. S33 shows the *J-V* curves of the fabricated devices on a double logarithmic scale, which comprises the Ohmic region, the trap-filling limit (TFL) region and the Child region. In the TFL region, the trap-state density (N_t) can be calculated by the following equation：

$$N_t = \frac{2\varepsilon\varepsilon_0 V_{TFL}}{qL^2} \quad \text{(S1)}$$

where ε and ε_0 are the relative dielectric constant and vacuum permittivity, respectively. V_{TFL} is the onset voltage of TFL region, q is elementary charge. L represents perovskite thin film thickness (shown in Fig. S32).

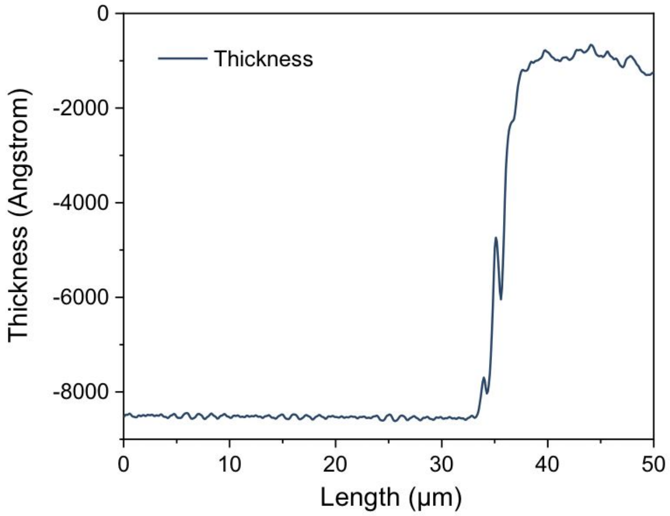

Fig. S31 Thickness measurement of perovskite films based on Dektak XTL

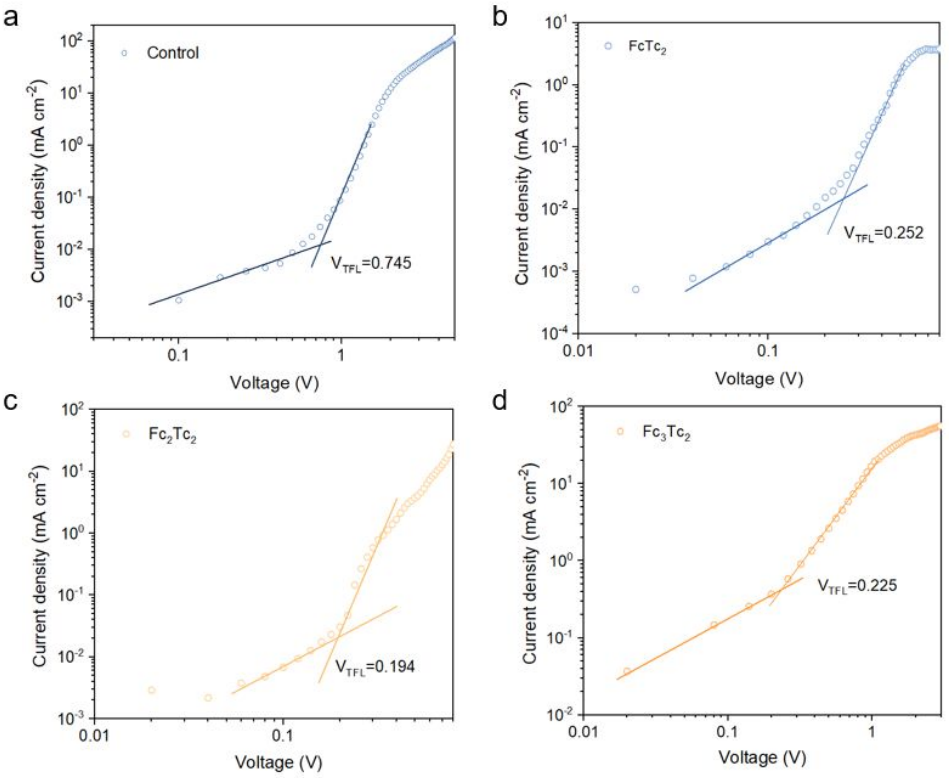

Fig. S32 Typical SCLC of defect density of the perovskite films (small-area) with different Fc compounds on electron-only device (FTO/TiO_2/perovskite/Fc/C60/BCP/Ag).

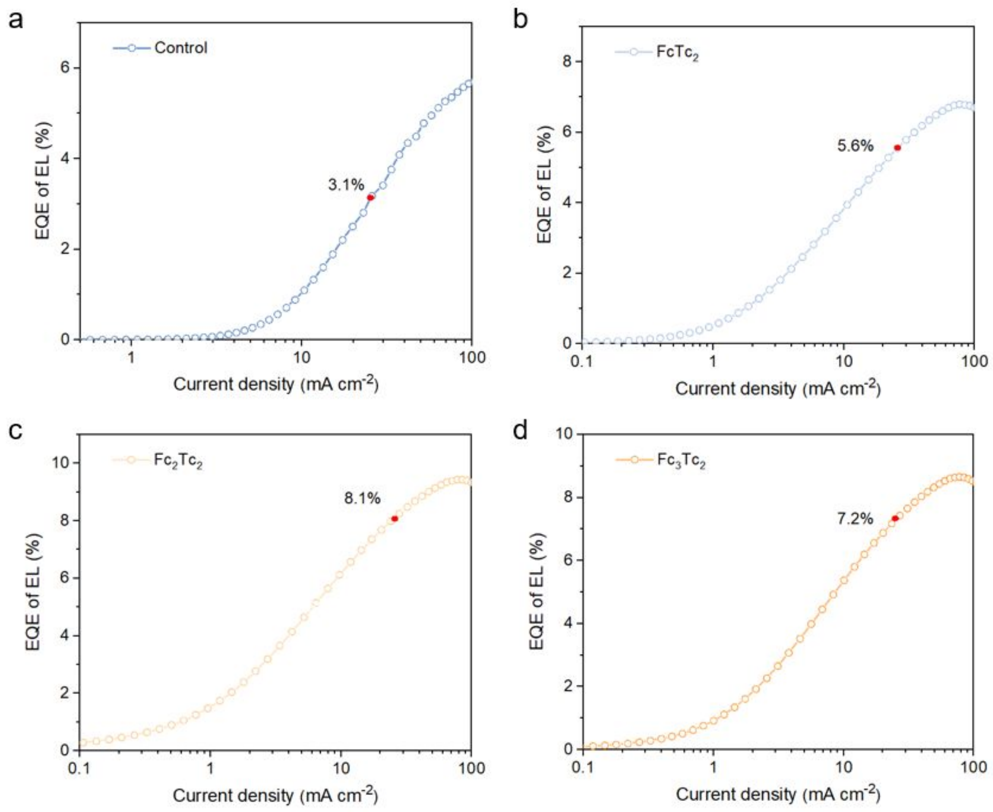

Fig. S33 Typical EQE of EL plots for the control, $FcTc_2$-, Fc_2Tc_2- and Fc_3Tc_2-treated PSCs.

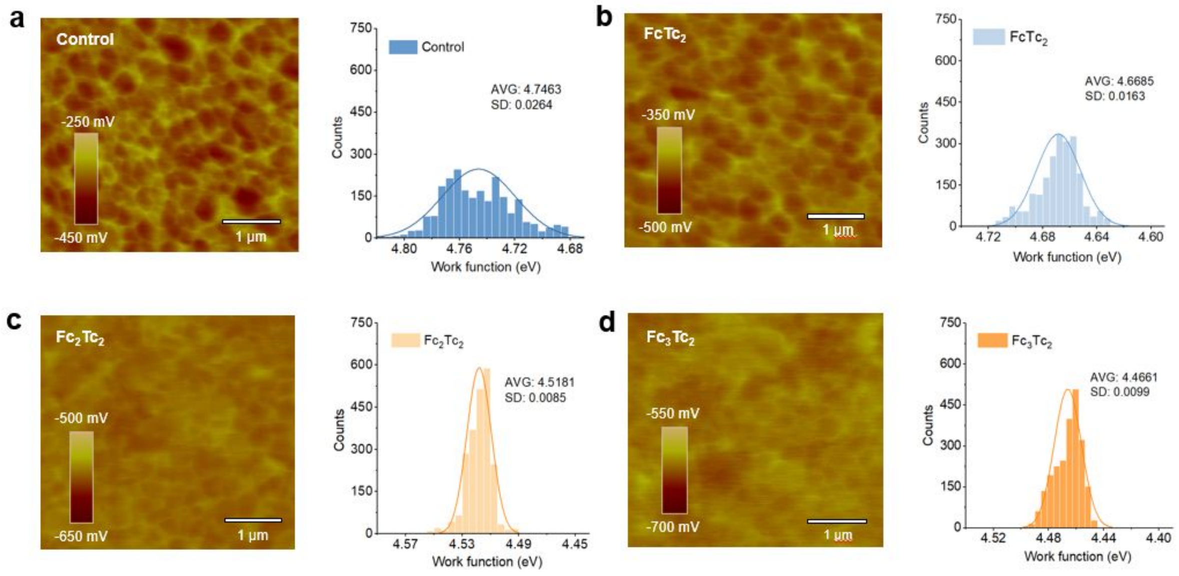

Fig. S34 KPFM images of contact potential difference (CPD) and the potential statistics of the perovskite films treated with different Fc molecules. The corresponding surface work functions of the perovskite films were calibrated by Au reference.

Note S7: PL map statistics.

The distribution of the PL map is obtained by counting the intensity of the 2D PL map image, as shown in Fig. S35. In order to test the extraction efficiency of carriers at the ETL/perovskite interface, we constructed the perovskite/Fc/C60 samples and performed PL mapping. We normalize the obtained PL intensity and unify it into an intensity range of 0-0.6, where 0 represents the minimum PL intensity. We extracted a total of 1500 positions (i.e., effective intensity values) from each PL map image, and finally performed intensity distribution statistics. In Fig. 2H, the distribution statistics of the PL map are presented in the form of a strip chart, where the horizontal axis represents the PL intensity and the vertical axis represents four groups of different samples. The color change of the strip chart represents the total number of points at different intensities, where yellow is the region where the intensity distribution is concentrated, and blue is the region where there is no intensity. The wider the yellow region, the more dispersed the intensity distribution.

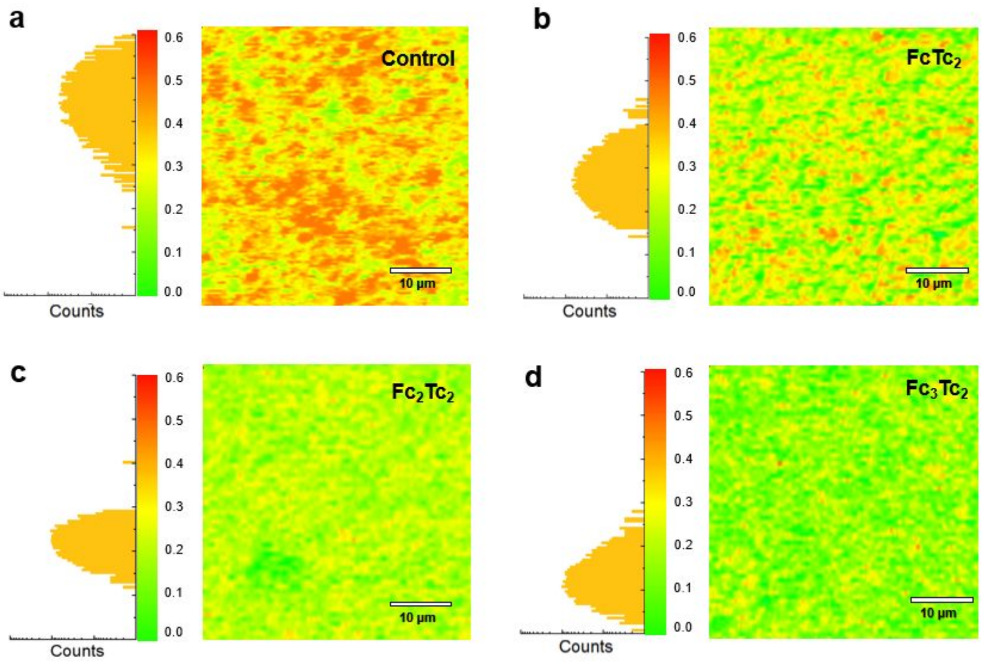

Fig. S35 PL mapping of the perovskite/Fc/C60 samples with a) control, b) $FcTc_2$-, c) Fc_2Tc_2- and d) Fc_3Tc_2-modification.

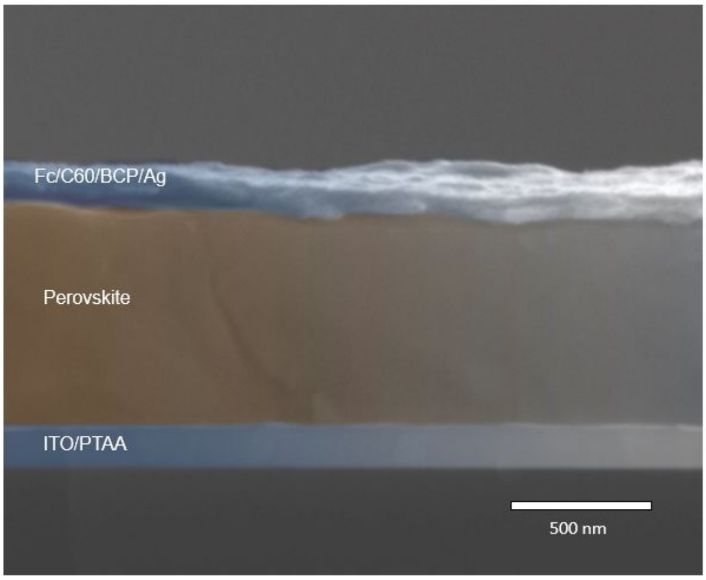

Fig. S36 Cross-section SEM image of Fc-modified inverted PSCs.

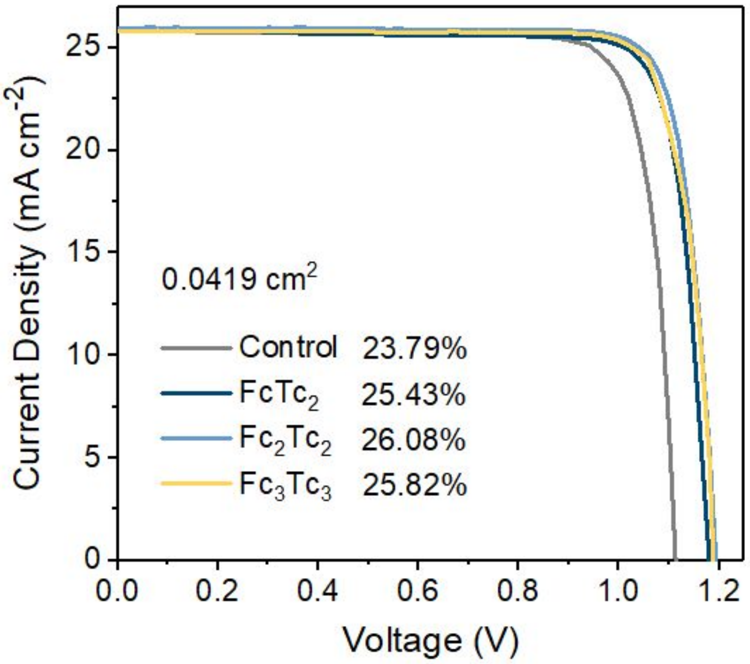

Fig. S37 *J-V* curves of the best-performing small-area PSCs modified with different Fc compounds.

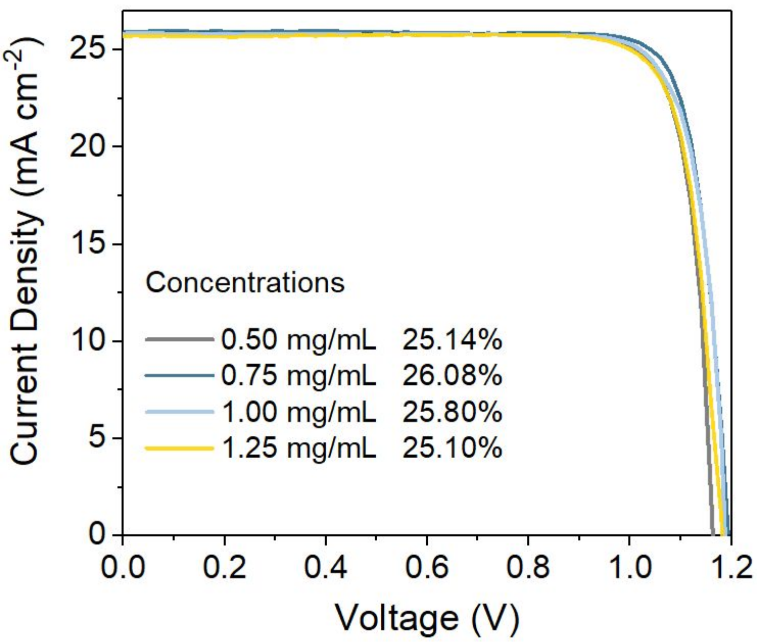

Fig. S38 *J-V* curves of the best-performance small-area PSCs modified with different concentrations of Fc_2Tc_2.

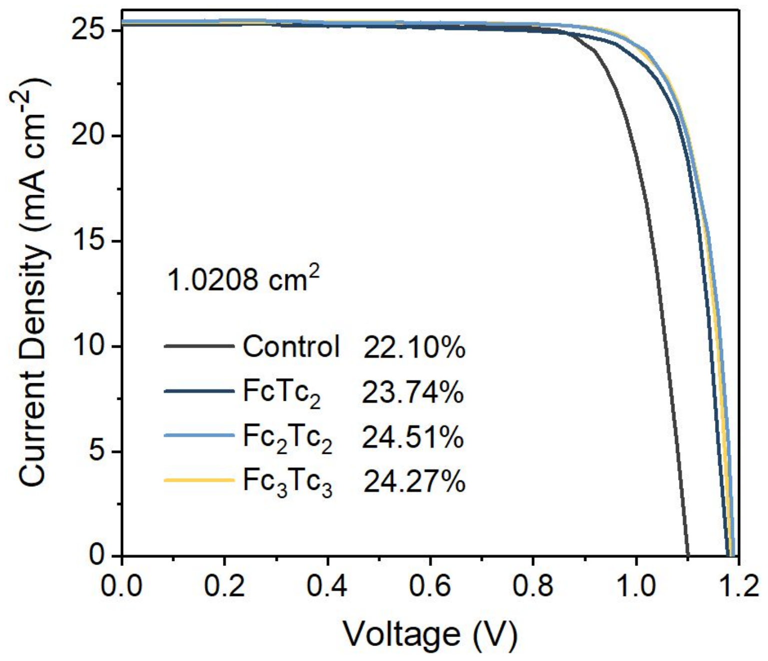

Fig. S39 *J-V* curves of the best-performing large-area PSCs modified with different Fc compounds.

中国认可
国际互认
检测
TESTING
CNAS L8490

Test and Calibration Center of New Energy Device and Module,
Shanghai Institute of Microsystem and Information Technology,
Chinese Academy of Sciences (SIMIT)

Measurement Report

Report No. 23TR072501

**Client Name**	Dr. Zhu Zonglong Research group
**Client Address**	83 Tat Chee Ave., City University of Hong Kong, Kowloon Tong, Hong Kong SAR
**Sample**	Perovskite Photovoltaic Cell
**Manufacturer**	City University of Hong Kong
**Measurement Date**	27th July, 2023

**Performed by:**	Qiang Shi *Qiang Shi*	**Date:** 27/07/2023
**Reviewed by:**	Wenjie Zhao *Wenjie Zhao*	**Date:** 27/07/2023
**Approved by:**	Yucheng Liu *Yucheng Liu*	**Date:** 14/08/2023

Address: No.235 Chengbei Road, Jiading, Shanghai **Post Code:**201800
E-mail: solarcell@mail.sim.ac.cn **Tel:** +86-021-69976921

The measurement report without signature and seal are not valid.
This report shall not be reproduced, except in full, without the approval of SIMIT.

Sample Information

Sample Type	Perovskite photovoltaic cel
Serial No.	8-1#
Lab Internal No.	23072501-1#
Measurement Item	I-V characteristic
Measurement Environment	24.3±2.0°C,44.3±5.0%R.H

Measurement of I-V characteristic

**Reference cell**	PVM 1121
**Reference cell Type**	mono-Si, WPVS, calibrated by NREL (Certificate No. ISO 2075)
**Calibration Value/Date of Calibration for Reference cell**	144.53mA/ Feb. 2023
**Measurement Conditions**	Standard Test Condition (STC):<br>Spectral Distribution: AM1.5 according to IEC 60904-3 Ed.3,<br>Irradiance: 1000±50W/m^2, Temperature: 25±2°C
**Measurement Equipment/ Date of Calibration**	AAA Steady State Solar Simulator (YSS-T155-2M) / July.2023<br>IV test system (ADCMT 6246) / June. 2023<br>SR Measurement system (CEP-25ML-CAS) / April.2023<br>Measuring Microscope (MF-B2017C) / July.2023
**Measurement Method**	I-V Measurement:<br>Logarithmic sweep in both directions (Voc to Isc and Isc to Voc) during one flash based on IEC 60904-1:2020;<br>Spectral Mismatch factor was calculated according to IEC 60904-7 and I-V correction according to IEC 60891;
**Measurement Uncertainty**	Area: 1.0%(k=2); Isc: 1.9%(k=2); Voc: 1.0%(k=2);<br>Pmax: 2.4%(k=2); Eff: 2.5%(k=2)

Report No. 23TR072501

====Measurement Results ====

	Forward Scan (Isc to Voc)	Reverse Scan (Voc to Isc)
Area	4.19 mm^2	
Isc	1.083 mA	1.084 mA
Voc	1.187 V	1.191 V
Pmax	1.067 mW	1.082 mW
Ipm	1.023 mA	1.034 mA
Vpm	1.043 V	1.047 V
FF	83.01 %	83.88 %
Eff	25.46 %	25.83 %

- Spectral Mismatch Factor: SMM=0.9960.
- Designated illumination area defined by a thin metal mask was measured by the measuring microscope.
- Test results listed in this measurement report refer exclusively to the mentioned measured sample.
- The results apply only at the time of the test, and do not imply future performance.

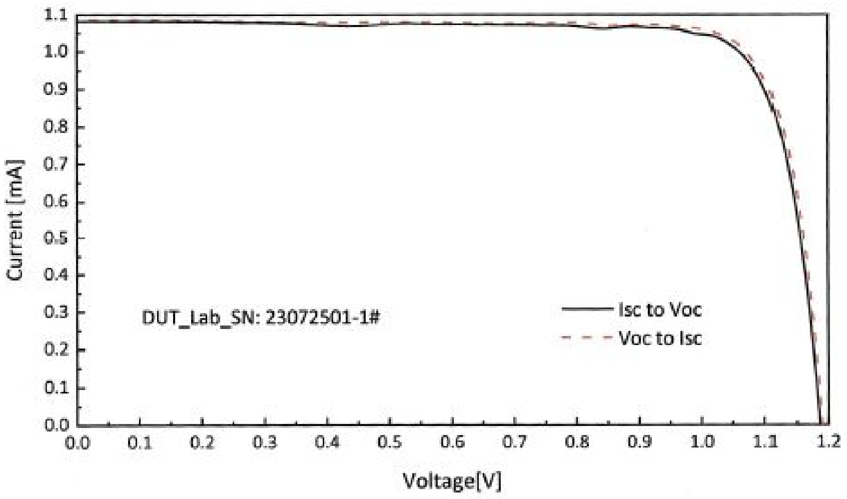

Fig.1 I-V curves of the measured sample

3 / 4

Fig. S40 Independent efficiency certification of small-area perovskite solar cells by an accredited institute of Shanghai Institute of Microsystem and Information Technology, Chinese Academy of Sciences (SIMIT), achieving a PCE of 25.83%, which is one of the highest values of the certified efficiency for inverted perovskite solar cells. This report is reproduced with the permission from SIMIT.

中国认可
国际互认
检测
TESTING
CNAS L8490

170017163974

Test and Calibration Center of New Energy Device and Module,
Shanghai Institute of Microsystem and Information Technology,
Chinese Academy of Sciences (SIMIT)

Measurement Report

Report No. 23TR041802

**Client Name**	Dr. Zhu Zonglong Research group
**Client Address**	83 Tat Chee Ave., City University of Hong Kong, Kowloon Tong, Hong Kong SAR
**Sample**	Perovskite Photovoltaic Cell
**Manufacturer**	City University of Hong Kong
**Measurement Date**	18th April, 2023

**Performed by:**	Qiang Shi Qiang shi	**Date:** 18/04/2023
**Reviewed by:**	Wenjie Zhao Wenjie Zhao	**Date:** 18/04/2023
**Approved by:**	Zhengxin Liu Zhengxin Liu	**Date:** 18/04/2023

Address: No.235 Chengbei Road, Jiading, Shanghai
Post Code:201800
E-mail: solarcell@mail.sim.ac.cn
Tel: +86-021-69976921

The measurement report without signature and seal are not valid.
This report shall not be reproduced, except in full, without the approval of SIMIT.

Sample Information

Sample Type	Perovskite photovoltaic cell
Serial No.	12-7#
Lab Internal No.	23041802-1#
Measurement Item	I-V characteristic
Measurement Environment	23.2±2.0°C, 43.7±5.0%R.H

Measurement of I-V characteristic

**Reference cell**	PVM1121
**Reference cell Type**	mono-Si, WPVS, calibrated by NREL (Certificate No. ISO 2075)
**Calibration Value/Date of Calibration for Reference cell**	144.53mA/ Feb. 2023
**Measurement Conditions**	Standard Test Condition (STC):<br>Spectral Distribution: AM1.5 according to IEC 60904-3 Ed.3,<br>Irradiance: 1000±50W/m^2, Temperature: 25±2°C
**Measurement Equipment/ Date of Calibration**	AAA Steady State Solar Simulator (YSS-T155-2M) / July.2022<br>IV test system (ADCMT 6246) / June. 2022<br>SR Measurement system (CEP-25ML-CAS) / April.2022<br>Measuring Microscope (MF-B2017C) / July.2022
**Measurement Method**	I-V Measurement:<br>Logarithmic sweep in both directions (Voc to Isc and Isc to Voc) during one flash based on IEC 60904-1:2006;<br>Spectral Mismatch factor was calculated according to IEC 60904-7 and I-V correction according to IEC 60891.
**Measurement Uncertainty**	Area: 1.0%(k=2); Isc: 1.9%(k=2); Voc: 1.0%(k=2);<br>Pmax: 2.4%(k=2); Eff: 2.5%(k=2)

Report No. 23TR041802

====Measurement Results ====

	Forward Scan (Isc to Voc)		Reverse Scan (Voc to Isc)	
Area	102.08 mm²			
Isc	25.861	mA	25.899	mA
Voc	1.183	V	1.184	V
Pmax	23.952	mW	24.265	mW
Ipm	24.418	mA	24.710	mA
Vpm	0.981	V	0.982	V
FF	78.30	%	79.16	%
Eff	23.46	%	23.77	%

- Spectral Mismatch Factor SMM=1.0029.
- Designated illumination area defined by a thin metal mask was measured by a measuring microscope.
- Test results listed in this measurement report refer exclusively to the mentioned test sample.
- The results apply only at the time of the test, and do not imply future performance.

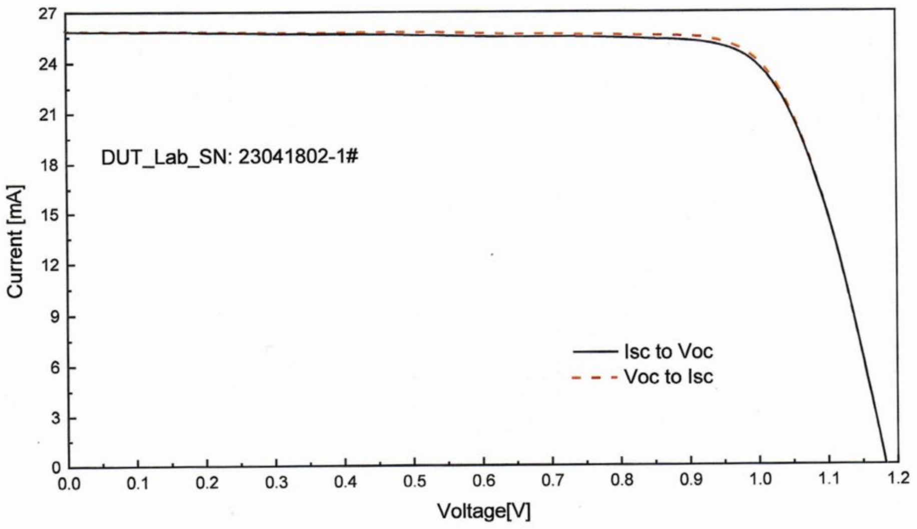

Fig.1 I-V curves of the measured sample

3 / 4

Fig. S41 Independent efficiency certification of large-area perovskite solar cells by an accredited institute of Shanghai Institute of Microsystem and Information Technology, Chinese Academy of Sciences (SIMIT), achieving a PCE of 23.77%, which is one of the highest certified efficiencies for centimeter-scale perovskite solar cells. This report is reproduced with the permission from SIMIT.

Note S8: Photovoltaic parameter statistics.

For the photovoltaic parameter statistics shown in Fig. S42, each parameter, including V_{OC}, J_{SC}, FF and PCE, was normalized to the highest value of the corresponding parameter. For example, for the PCE, the highest value appears in the Fc_2Tc_2 (Fc_2.) treated small-area device, and all PCEs in control small, control large, Fc_2. small, and Fc_2. large were normalized to is the highest value of the Fc_2. treated small-area device. For the parameter increase rate in Fig. 3D and 3E, the average values of parameters are calculated. Specifically, the parameter of the Fc_2. minus the parameter of the control, and then divides it by the parameter of the control to obtain the increase rate. For the loss rate, the parameter of the large-area device is subtracted from the parameter of the small-area device (absolute value), and then divided by the parameter of the small-area device to obtain the reduction rate.

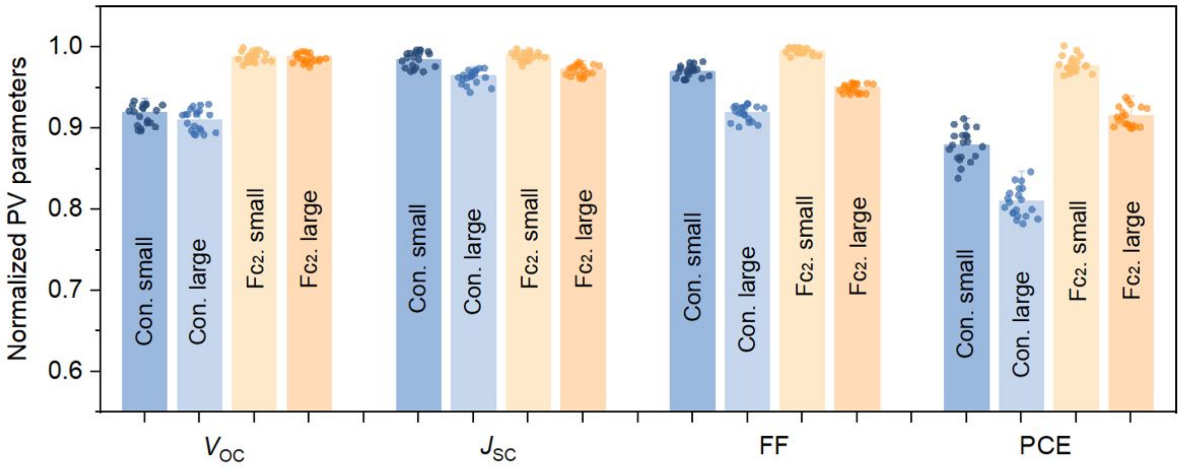

Fig. S42 Statistics of the normalized photovoltaic parameters for 20 individual small- and large-area devices with and without Fc_2Tc_2. The scatter points represent the parameters of each device, and the histogram is the average value. Data are normalized to the highest value of each parameter.

Note S9: Calculation of QFLS

Assuming that all PL emission comes from the radiative recombination of free charges in the perovskite film, PLQY is directly related to QFLS and can be expressed by the following equation.

$$QFLS = K_B T ln(PLQY \times J_G/J_{0,rad}) \qquad (S2)$$

where K_B, and T represent Boltzmann constant and temperature, respectively. J_G is the current density under illumination, and $J_{0,rad}$ is the dark radiative recombination saturation current density.

The following equations can be used to calculate the in accordance with the detailed balance theory:

$$J_{0,rad} = q\int_0^{\infty} EQE_{PV}(E)\phi_{BB}(E)dE \qquad (S3)$$

$$\phi_{BB}(E) = \frac{2\pi E^2}{h^3c^2}\frac{1}{\exp\left(\frac{E}{k_BT}\right) - 1} \qquad (S4)$$

Where q, EQE_{PV}, ϕ_{BB}, E, h, c represents elementary charge, photovoltaic external quantum efficiency, black-body radiative spectrum, photon energy, Planck constant, and light speed in vacuum, respectively.

The highly-sensitive EQE shown in Fig. S44, which can be applied to evaluate the $J_{0,rad}$. The $J_{0,rad}$ can be calculated as 5.42×10^{-21} mA cm^{-2} for the control device, and 1.17×10^{-21} mA cm^{-2} for the Fc_2Tc_2 treated device. In this instance, J_G stands for the generation current density under illumination, which is roughly equivalent to the J_{SC} of devices. Subsequently, the PLQY of samples were measured with a 375 nm laser inside an integrated sphere, which are 0.00145 and 0.00423 for the control and Fc_2Tc_2 treated device, respectively. The laser illuminates the samples with a 1-sun equivalent intensity by adjusting the produced current close to the J_{SC} of the devices. Then the QFLS of samples can be calculated from equation S2.

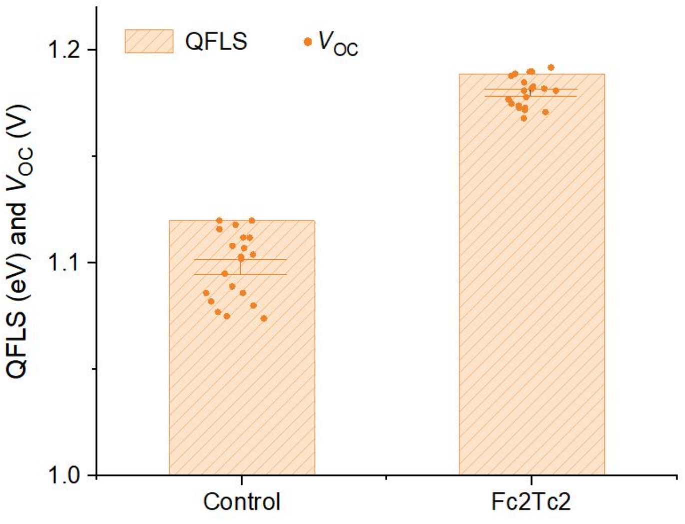

Fig. S43 Comparison of the V_{OC} of PSCs with the QFLS of representative layer stacks.

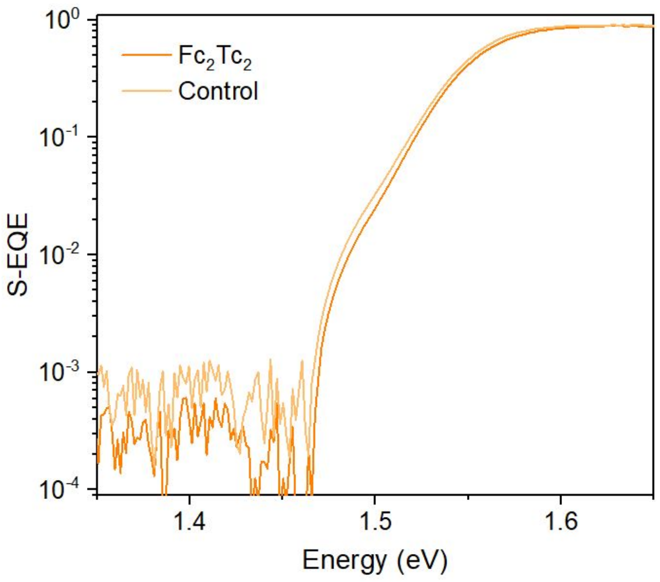

Fig. S44 The highly sensitive EQE of PSCs with and without Fc_2Tc_2.

Note S10: Light intensity dependent open-circuit voltage measurements.

The ideality factor (n) can be extracted according to $V_{OC}(P)$ = nkT/q·ln(P) + C, where T is the absolute temperature, P is the incident light intensity, q is the elementary charge, C is a constant, k is the Boltzmann constant, and T is the absolute temperature. In general, an ideality factor of 1 is associated to bimolecular bond-to-bond radiative recombination of carriers or dominating Shockley–Read–Hall (SRH) trap-assisted recombination with one pinned charge carrier density, while an ideality factor of 2 is associated with dominated SRH recombination without pinning of one charge carrier density. In Fig. S45, the measurements were conducted on three individual devices and the n value is the average value of three devices.

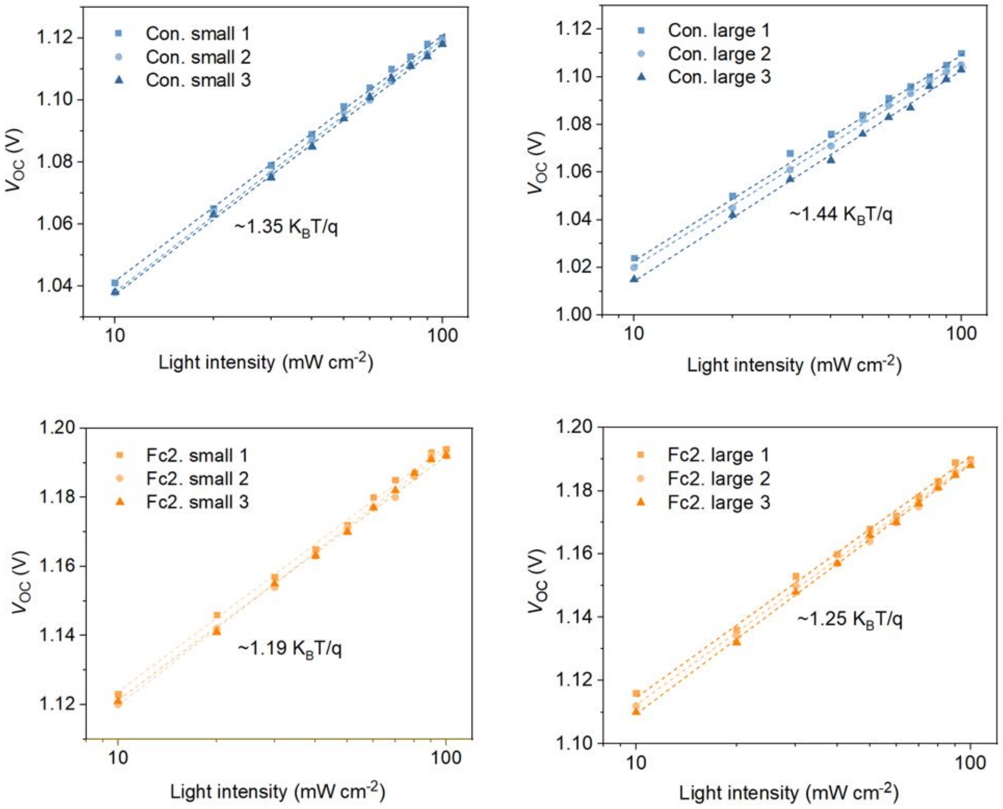

Fig. S45 Light intensity dependent voltages for large- and small-area PSCs with and without Fc_2Tc_2 treatment.

Note S11: FF loss calculation.

The FF loss between the Shockley–Queisser limit and measured FF value is composed of non-radiative loss and resistance loss, and the maximum FF (FF_{max}) can be empirically calculated with the equation:

$$FF_{max}= \frac{\nu OC - \ln(\nu OC + 0.72)}{\nu OC + 1} \quad (S5)$$

, where $v_{OC}=qV_{OC}/nk_BT$, q denotes elementary charge, Voc is open-circuit voltage, n is ideality factor, k_B is Boltzmann constant and T is temperature. The ideality factor n is obtained from the Note S10 and Fig. S45. The non-radiative recombination loss is equal to the difference between $FF_{SQ\text{-}limit}$ and FF_{max}, and the transport loss is equal to the difference between FF_{max} and FF values of devices.

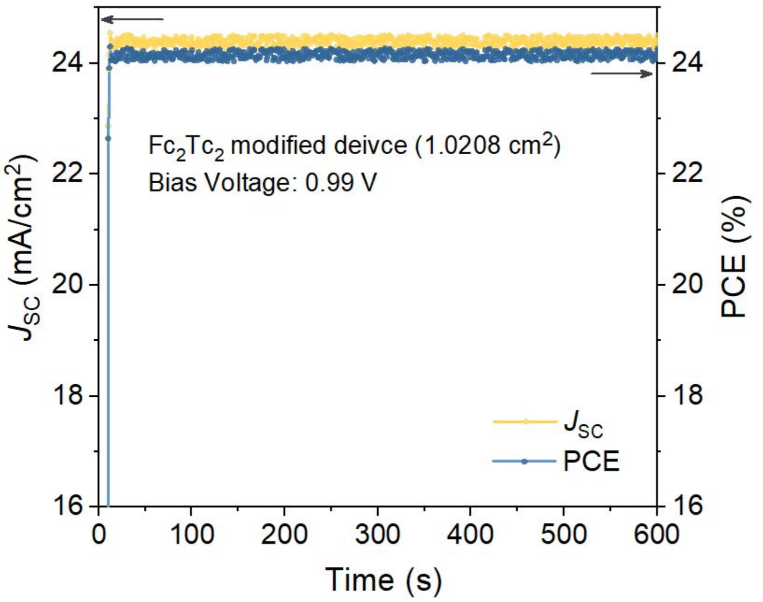

Fig. S46 MPP of the best-performing large-area PSCs modified with Fc_2Tc_2.

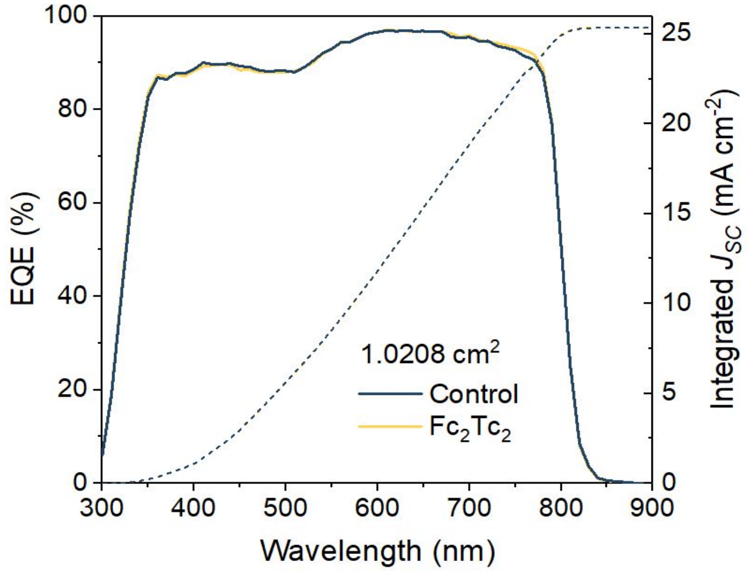

Fig. S47 EQE curves and integrated J_{SC} for large-area devices.

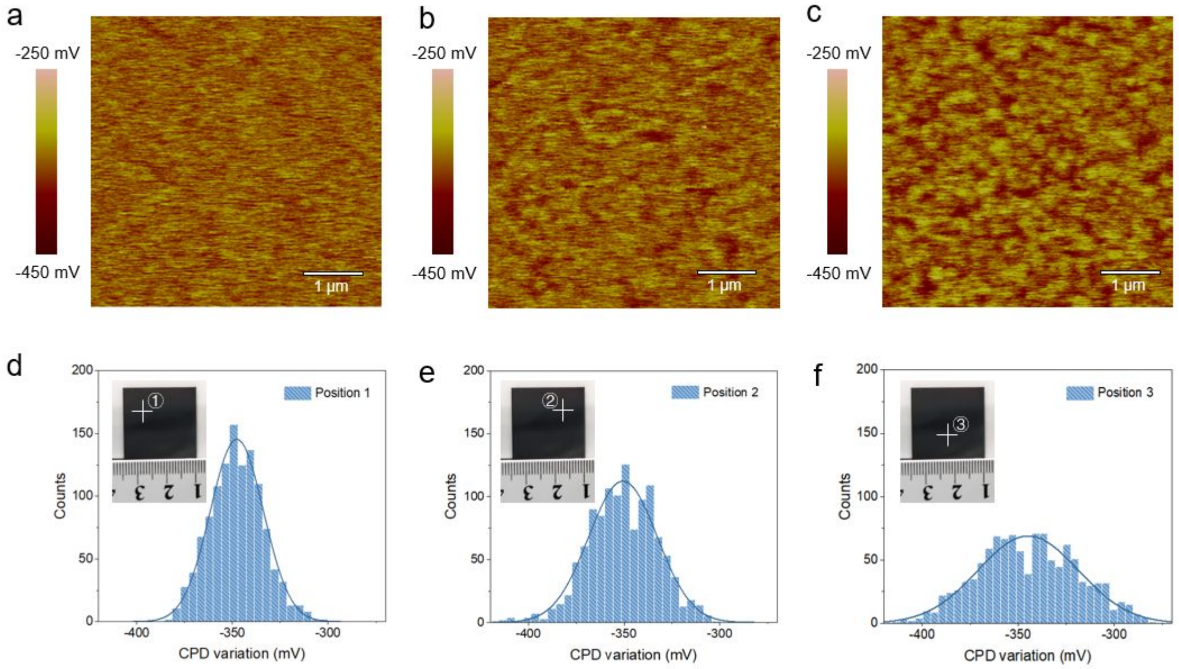

Fig. S48 KPFM image and potential distribution of control films at three different sites.

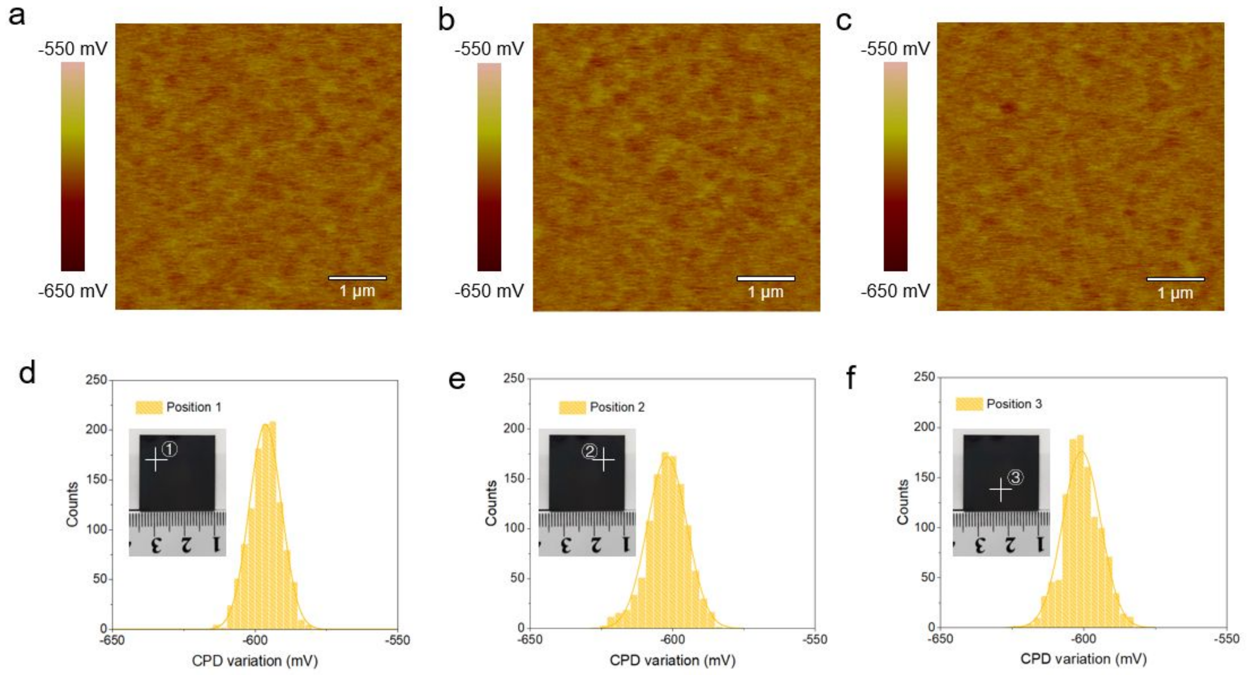

Fig. S49 KPFM image and potential distribution of Fc_2Tc_2-treated films at three different sites.

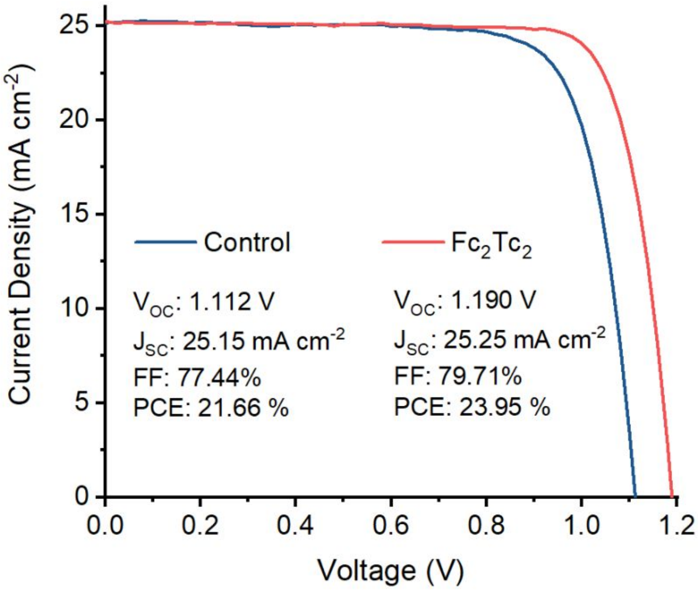

Fig. S50 Initial efficiencies of the large-area devices before MPPT testing

Table S3 A summary of the state-of-the-art results reported in the literature for the simultaneous fabrication of large- and small-area PSCs.

Modulation	Strategy	Device	FF (%)	V_{oc} (V)	PCE (%)	Ref.
Bulk perovskite	Hydrogen-bond-bridged intermediate phase	Small	84.78	1.19	24.80	12
		Large	77.43	1.18	22.70	
	MDA cation replacement	Small	82.15	1.168	25.17	13
		Large	73.8	1.167	21.6	
	Liquid medium annealing technology	Small	81.18	1.151	23.24	14
		Large	78.54	1.155	22.17	
	Ordered dipolar for crystallization modulation	Small	84.3	1.18	24.60	15
		Large	80.7	1.18	23.10	
Transport layer	Two fluorinated isomeric analogs HTL	Small	80.90	1.164	24.82	16
		Large	74.22	1.178	22.31	
	Improvements in charge carrier management	Small	84.7	1.194	25.4	17
		Large	79.5	1.196	22.7	
	Polyacrylic acid–stabilized tin(IV) oxide quantum dots	Small	83.8	1.18	25.7	18
		Large	79.1	1.16	23.3	
	Self-assembled monolayer with NiO nanocrystal film	Small	78.3	2.00	24.7	19
		Large	74.1	2.02	23.5	
Interface	Sulfurization for building heterojunctions	Small	82.9	1.19	24.30	20
		Large	70.8	1.17	20.70	
	0D zinc halogenometallate passivation	Small	83.9	1.12	24.10	21
		Large	78.4	1.13	20.80	
	Polymer template for “Mortise-Tenon” structure	Small	80.2	1.2	24.69	22
		Large	79.3	1.19	23.06	
	Superwetting underlayer for deposition and passivation	Small	84.7	1.21	25.4	23
		Large	80.9	1.19	23.4	
**Interface**	**Fc-induced electronic homogenization**	**Small**	**84.24**	**1.194**	**26.08**	**This work**
		**Large**	**80.82**	**1.190**	**24.51**	

Table S4 Statistics of the trap-filling voltage V_{TFL} for perovskite devices with different Fc compounds.

Sample	V_{TFL} (V)	Sample	V_{TFL} (V)	Sample	V_{TFL} (V)	Sample	V_{TFL} (V)
Con.	0.745	$FcTc_2$	0.252	Fc_2Tc_2	0.194	Fc_3Tc_2	0.225
	0.753		0.263		0.199		0.235
	0.816		0.342		0.203		0.226
	0.854		0.352		0.263		0.260
	0.823		0.410		0.203		0.287
	0.950		0.374		0.221		0.254
	0.934		0.283		0.283		0.238
	0.851		0.433		0.270		0.284
	0.873		0.365		0.254		0.299

Table S5 Statistics of the EQE-EL values for perovskite devices with different Fc compounds.

Sample	EQE_{EL} (%)	Sample	EQE_{EL} (%)	Sample	EQE_{EL} (%)	Sample	EQE_{EL} (%)
Con.	3.1	$FcTc_2$	5.6	Fc_2Tc_2	8.1	Fc_3Tc_2	7.2
	2.3		5.5		7.9		6.7
	2.4		4.9		8.0		6.5
	2.6		5.2		8.1		6.3
	3.0		5.5		7.7		6.5
	2.3		4.9		7.5		7.0

Table S6. Fitting results of TRPL transients for perovskite films. Bi-exponential fitting method with the equation $y(t) = A_1\exp(-t/\tau_1)+A_2\exp(-t/\tau_2)+y_0$ was used. The average lifetime can be calculated with the equation $\tau_{avg} = (A_1\tau_1^2 + A_2\tau_2^2)/(A_1\tau_1 + A_2\tau_2)$.

Sample	A_1	τ_1 (ns)	A_2	τ_2 (ns)	τ_{avg} (ns)
Control	0.55	8.87	0.56	109.86	102.44
$FcTc_2$	2.08	7.60	0.29	102.52	69.57
Fc_2Tc_2	59.22	2.26	0.65	27.84	5.31
Fc_3Tc_2	54.52	2.83	0.64	24.74	4.87

Table S7 Photovoltaic parameters of best-performing small-area PSCs modified with different functional Fc molecules.

Sample	V_{OC} (V)	J_{SC} (mA cm^{-2})	FF (%)	PCE (%)
Control	1.113	25.82	82.78	23.79
$FcTc_2$	1.180	25.80	83.54	25.43
Fc_2Tc_2	1.194	25.93	84.24	26.08
Fc_3Tc_2	1.189	25.83	84.07	25.82

Table S8 Photovoltaic parameters of best-performing small-area PSCs with different excess PbI_2 and different concentrations of Fc_2Tc_2 modification.

Excess PbI_2 (mol%)	Fc concentration (mg mL^{-1})	V_{OC} (V)	J_{SC} (mA cm^{-2})	FF (%)	PCE (%)
0	0.50	1.157	25.90	83.31	24.96
	0.75	1.179	25.92	83.51	25.52
	1.00	1.176	25.81	83.95	25.48
	1.25	1.171	25.76	82.03	24.74
2	0.50	1.161	25.83	83.83	25.14
	0.75	1.194	25.93	84.24	26.08
	1.00	1.187	25.84	84.13	25.80
	1.25	1.181	25.71	82.68	25.10
5	0.50	1.165	25.42	83.48	24.72
	0.75	1.192	25.47	83.90	25.47
	1.00	1.190	25.52	84.17	25.56
	1.25	1.174	25.29	81.94	24.33

Table S9 Photovoltaic parameters of best-performing large-area PSCs modified with different functional Fc molecules.

	V_{OC} (V)	J_{SC} (mA cm^{-2})	FF (%)	PCE (%)
Control	1.110	25.30	78.68	22.10
$FcTc_2$	1.180	25.34	79.36	23.74
Fc_2Tc_2	1.190	25.48	80.82	24.51
Fc_3Tc_2	1.185	25.42	80.56	24.27

Table S10 Small-area photovoltaic parameters captured at different locations of the 1 cm^2 device

Position	V_{OC} (V)	J_{SC} (mA cm^{-2})	FF (%)	PCE (%)
1	1.172	25.53	82.95	24.82
2	1.175	25.57	82.11	24.67
3	1.176	25.39	82.82	24.73
4	1.171	25.53	82.79	24.75
5	1.172	25.41	82.87	24.68

References

1. M. S. Inkpen *et al.* Oligomeric ferrocene rings. *Nat. Chem.* 2016, **8**, 825-830.
2. Z. Li *et al.* Organometallic-functionalized interfaces for highly efficient inverted perovskite solar cells. *Science* 2022, **376**, 416-420.
3. O. V. Dolomanov et al. OLEX2: a complete structure solution, refinement and analysis program. *J. Appl. Crystallogr.* 2009, **42**, 339-341.
4. G. Sheldrick, SHELXTL, V5. 1, Bruker AXS, Madison, Wisconsin, 1998. *Received February* 2002, **14**, I02078.
5. G. Sheldrick, Crystal structure refinement with SHELXL. *Acta Crystallographica Section C* 2015, **71**, 3-8.
6. G. Kresse, Ab-Initio Molecular-Dynamics for Liquid-Metals. *J. Non-Cryst. Solids* 1995, **193**, 10239–10265.
7. P. E. Blöchl, Projector Augmented-Wave Method. *Phys. Rev. B* 1994, **50**, 17953–17979.
8. J. P. Perdew et al. Generalized Gradient Approximation Made Simple. *Phys. Rev. Lett.* 1996, **77**, 3865–3868.
9. S. Grimme, Semiempirical GGA-type density functional constructed with a long-range dispersion correction. *J. Comp. Chem.* 2006, **27**, 1787–99.
10. S. Grimme et al. A consistent and accurate ab initio parametrization of density functional dispersion correction (DFT-D) for the 94 elements H-Pu. *J. Chem. Phys.* 2010, **132**, 154104.
11. J. Moellmann et al. DFT-D3 Study of Some Molecular Crystals. *J. Phys. Chem. C* 2014, **118**, 7615–7.
12. F. Li et al. Hydrogen-bond-bridged intermediate for perovskite solar cells with enhanced efficiency and stability. *Nat. Photon.* 2023, **17**, 478–484.
13. G. Kim et al., Impact of strain relaxation on performance of α-formamidinium lead iodide perovskite solar cells. *Science* 2020, **370**, 108-112.
14. N. Li et al., Liquid medium annealing for fabricating durable perovskite solar cells with improved reproducibility. *Science* 2021, **373**, 561-567.
15. G. Li et al. Highly efficient p-i-n perovskite solar cells that endure temperature variations. *Science* 2023, **379**, 399-403.
16. M. Jeong et al., Stable perovskite solar cells with efficiency exceeding 24.8% and 0.3-V voltage loss. *Science* 2020, **369**, 1615-1620.
17. J. J. Yoo et al., Efficient perovskite solar cells via improved carrier management. *Nature 2021,* **590**, 587-593.
18. M. Kim et al. Conformal quantum dot–SnO_2 layers as electron transporters for efficient perovskite solar cells. *Science* 2022, **375**, 302-306.
19. L. Li. et al. Flexible all-perovskite tandem solar cells approaching 25% efficiency with molecule-bridged hole-selective contact. *Nat. Energy*, 2022, **7**, 708–717.
20. X. Li et al. Constructing heterojunctions by surface sulfidation for efficient inverted perovskite solar cells. *Science* 2022, **375**, 434-437.
21. S. Ye et al. Expanding the low-dimensional interface engineering toolbox for efficient perovskite solar cells. *Nat Energy* 2023, **8**, 284–293.
22. F. Wang et al. Monolithically-grained perovskite solar cell with Mortise-Tenon structure for charge extraction balance. *Nat. Commun.* 2023, **14**, 3216.

23. S. Zhang et al. Minimizing buried interfacial defects for efficient inverted perovskite solar cells. *Science* 2023, **380**, 404-409.
